# Author Correction: Mapping in situ the assembly and dynamics in aqueous supramolecular polymers

**DOI:** 10.1038/s41467-026-72528-z

**Published:** 2026-04-24

**Authors:** Huachuan Du, Ruomeng Qiu, Xianwen Lou, Stef A. H. Jansen, Hiroaki Sai, Yuyang Wang, Albert J. Markvoort, E. W. Meijer, Samuel I. Stupp

**Affiliations:** 1 Center for Regenerative Nanomedicine, Northwestern University, Chicago, IL USA; 2 Institute for Complex Molecular Systems, Eindhoven University of Technology, Eindhoven, The Netherlands; 3 Department of Chemistry and Chemical Engineering and Laboratory of Macromolecular and Organic Chemistry, Eindhoven University of Technology, Eindhoven, The Netherlands; 4 Department of Chemistry, Northwestern University, Evanston, IL USA; 5 Department of Applied Physics and Science Education, Eindhoven University of Technology, Eindhoven, The Netherlands; 6 Department of Biomedical Engineering and Synthetic Biology Group, Eindhoven University of Technology, Eindhoven, The Netherlands; 7 School of Chemistry and RNA Institute, University of New South Wales, Sydney, NSW Australia; 8 Department of Medicine, Northwestern University, Chicago, IL USA; 9 Department of Biomedical Engineering, Northwestern University, Evanston, IL USA; 10 Department of Materials Science and Engineering, Northwestern University, Evanston, IL USA

Correction to: *Nature Communications* 10.1038/s41467-025-60138-0, published online 24 May 2025

Although the impact of carbonate on the pH-titrations in this article has been minimized by quantifying the carbonate level, the carbonate levels are low and likely variable. Therefore, a small variability of the pH could not be excluded. The authors therefore want to make clear that any contribution from carbonate levels has been neglected and that this may impact the precise values of Hill coefficients, without changing the conclusion that the deprotonation process is cooperative.

In the corrected version of the article the Authors state “It is important to note that the presence of a small amount of carbonate is not considered in the calculation of the Hill coefficients and this may have an impact on the calculated values; as a result, the deprotonation process of the peptide amphiphiles micelles inferred from the Hill analysis may be slightly under- or overestimated” and the expression “highly cooperative” has been changed to “cooperative” in the first paragraph of the Discussion section.

The changes are made in the PDF and HTML versions of the article.

