# Supplementary material for: Mapping in situ the assembly and dynamics in aqueous supramolecular polymers
Source: Nat Commun. 2025 May 24;16:4837. doi: 10.1038/s41467-025-60138-0 (PMC12103577; doi:10.1038/s41467-025-60138-0)
Supplement: Supplementary file 1 — Supplementary Information [file 41467_2025_60138_MOESM1_ESM.pdf]

Supplementary Information for

Mapping *In Situ* the Assembly and Dynamics in Aqueous Supramolecular Polymers

Huachuan Du, Ruomeng Qiu, Xianwen Lou, Stef A. H. Jansen, Hiroaki Sai, Yuyang Wang, Albert J. Markvoort, E. W. Meijer, and Samuel I. Stupp
Corresponding author: s-stupp@northwestern.edu and e.w.meijer@tue.nl

The PDF file includes:

Supplementary Text
Supplementary Fig. 1 to 57
Supplementary Table 1 to 17
References

Supplementary Text

1. *m*-factor mass-balance model

By analogy to protein unfolding studies[1], we derived our first thermodynamic model to depict the transformation of monomers within micelles (M) into those within polymers (P) as,

$$M \underset{}{\overset{K}{\rightleftharpoons}} P \tag{S1}$$

where K corresponds to the apparent equilibrium constant for this transformation. Therefore, the corresponding apparent Gibbs free energy change (ΔG) can be expressed as,

$$\Delta G = R \cdot T \cdot \ln K = R \cdot T \cdot ln \frac{[P]}{[M]}. \tag{S2}$$

By assuming the activity of the monomers within micelles and polymers is equal to their concentrations, we obtained,

$$\frac{[P]}{[M]} = \frac{\phi_{\beta-\text{sheets}}}{1-\phi_{\beta-\text{sheets}}} \tag{S3}$$

Hence, we could extract ΔG from the molar fraction of monomers within β-sheet polymers ($\phi_{\beta-\text{sheets}}$) obtained from our CD titration curves,

$$\Delta G = R \cdot T \cdot ln \frac{\phi_{\beta-\text{sheets}}}{1-\phi_{\beta-\text{sheets}}}. \tag{S4}$$

The resulting ΔG curves appeared to be linearly dependent on the titrated [NaOH]/[PA] in the micelle-to-polymer transformation region, as shown in Supplementary Fig. 25a. Leveraging this observation, a constant ΔG_0 and concentration-dependent *m* factors were introduced as variables to predict the values of ΔG,

$$\Delta G = \Delta G_0 + m \frac{[NaOH]}{[PA]} \tag{S5}$$

The equilibrium model was fitted to the $\phi_{\beta\text{-sheets}}$ of experimental titration curves using least-squares minimization. The difference between all experimentally obtained datapoints and the corresponding simulated values were combined in a cost vector. The minimization of the cost vector was performed using the Matlab® lsqnonlin function with the Levenberg-Marquardt algorithm. To ensure that the solution is at the global minimum, the fits were performed with 500 initial parameter sets. The initial parameter sets were defined using a Latin Hypercube Sampling method, implemented with the Matlab® function lhsdesign. To ensure reasonable values of the set of starting parameters in the fitting of the melting curves, ΔG_0 was sampled between 0 and 30 kJ/mol and *m* factors were sampled between –20 and –5 kJ/mol. An independent *m* factor was fitted for each concentration. The final parameter set that resulted in the lowest norm of the residual cost vector was selected as the best fit.

2. Allosteric binding model

Drawing inspiration from the ligand-receptor allosteric binding in biological signaling processes[2,3], we developed the second thermodynamic model to describe the cooperative deprotonation process of PA micelles. The sequential deprotonation of a fully protonated micelle composed of j monomers can be approximately represented by a series of addition of hydroxide ion (OH^-) to the initial micelle (M^{3j+}),

$$M^{3j+} + OH^- \overset{K_1}{\rightleftharpoons} M^{(3j-1)+} + H_2O$$

$$\vdots$$

$$M^{(3j-i+1)+} + OH^- \overset{K_i}{\rightleftharpoons} M^{(3j-i)+} + H_2O \quad \text{(S6)}$$

$$\vdots$$

$$M^{+} + OH^- \overset{K_{3j}}{\rightleftharpoons} M^{0} + H_2O$$

with K_i corresponding to the i-th binding constant of OH^- to the positively charged micelle species. Accordingly, a series of cooperative parameters (α) are defined as,

$$\alpha = K_{i+1}/K_i \quad \text{(S7)}$$

The apparent binding constant (K') for the deprotonation process is defined as geometric mean of individual binding constants,

$$K' = \sqrt[3j]{K_1 K_2 \cdots K_{3j}} \quad \text{(S8)}$$

Assuming identical cooperative parameter α for each deprotonation step, Supplementary Equation S8 can be simplified as,

$$K' = \sqrt[3j]{\alpha^{\sum_{i=1}^{3j-1} i}}\, K_1 \quad \text{(S9)}$$

In the classic allosteric binding of monovalent ligand (B) to receptors ($A_1 A_2 \ldots . A_n$) with multiple binding sites (n), the dependence of the binding site occupancy (θ_A) on the ligand concentration of ligand ($[B]$) can be generally approximated as[2],

$$\theta_A \approx \frac{(K'[B])^n}{1+(K'[B])^n} \quad \text{(S10)}$$

Similarly, we considered the deprotonated micelles species ranging from $M^{(3j-1)+}$ to M^0 as the occupied binding sites by titrated OH^-. By assuming all the monomers within these deprotonated micelle species as the effective ones to form β-sheet polymers, regardless of their molecular transformation pathway, the dependence of $\phi_{\beta\text{-sheets}}$ on the $[OH^-]$ can be expressed as,

$$\phi_{\beta\text{-sheets}} \approx \frac{(K'[OH^-])^{3j}}{1+(K'[OH^-])^{3j}} \quad \text{(S11)}$$

Indeed, if we consider a complete picture of the PA solution, the titrated NaOH could also bind H^+ ions governed by the dissociation equilibrium of water and carbonate (CO_3^{2-}) or bicarbonate ions (HCO_3^-) governed by the dissociation equilibrium of carbonic acid (H_2CO_3) due to the presence of dissolved atmospheric CO_2. This makes it difficult to precisely extract the $[OH^-]$ involved in the above deprotonation equilibria of micelles. Because the initial pH values of PA solutions (Supplementary Fig. 3a) and NaOH solutions (Supplementary Fig. 3b) are consistent

for the titration experiments of all three PA systems, we assume $[OH^-] = [NaOH]$ by neglecting the influence of the dissociation equilibrium of water and carbonic acid here.

Consequently, like the classic allosteric binding models, the cooperativity of PA supramolecular polymerization system was also represented in the Hill plot, where a rearrangement of Supplementary Equations S11 was carried out as,

$$log \frac{\phi_{\beta-\text{sheets}}}{1-\phi_{\beta-\text{sheets}}} = 3j \log(K'[NaOH]) \quad \text{(S12)}$$

The values of K' were obtained by substituting the $\phi_{\beta-\text{sheets}} = 0.5$ and the corresponding $[NaOH]$ extracted from the normalized CD titration curves as,

$$K' = 1/[NaOH]_{\phi_{\beta-\text{sheets}}=0.5} \quad \text{(S13)}$$

By plotting $log \frac{\phi_{\beta-\text{sheets}}}{1-\phi_{\beta-\text{sheets}}}$ as a function of $\log(K'[NaOH])$, the Hill coefficient (n_{H}) corresponding to the slope of this plot measured at $\phi_{\beta-\text{sheets}}$=0.5 can be a useful macroscopic parameter to quantify the cooperativity of deprotonation process for PA systems. The resulting Hill plots for all three PA homopolymerization systems display similar Hill coefficients (n_{H}) of approximately 10 (Fig. 3g, Supplementary Fig. 28 and 29). A Hill coefficient significantly larger than 1 strongly suggests a highly cooperative deprotonation process.[3]

Additionally, n_{H} is equivalent to the number of deprotonation steps and therefore provides an estimation of the micelle size. Each monomer contains three ionizable amine groups, leading to three potential final charged states of micelles: M^{2j+}, M^{j+}, or M^0, where 1, 2, or 3 amine groups per monomer are deprotonated, respectively. Consequently, the corresponding number of deprotonation steps would be *j*, *2j*, or 3*j*, all of which are equivalent to n_{H}. In our model, we assumed an extreme case where all monomers within the micelle must be fully deprotonated (M^0) during the assembly, which resulted in an estimated micelle size of approximately 3 monomers. Importantly, while it remains experimentally challenging to identify the exact charged state of micelles, the extraction of *K'* is independent of whether the final charged state is assumed to be M^{2j+}, M^{j+}, or M^0. The only difference lies in the implied micelle size: approximately 5 monomers in the case of M^{2j+} and 10 monomers in the case of M^{j+}.

3. H/D exchange mechanism for PA systems

The basic procedure in HDX-MS measurement involves diluting PA samples into D_2O by a factor of 100 to allow the H/D exchange of labile hydrogen atoms (−NH and $-NH_2$). To exclude the dilution-induced depolymerization, the three PA polymer samples were formed at [PA] = 4 mM and [NaOH]/[PA] = 15, whereas the PA-3 micelle sample was formed at [PA] = 4 mM and [NaOH]/[PA] = 0. Under the guidance of polymerization landscape, as shown in Fig. 2d and Supplementary Fig. 7, these samples are expected to remain as polymers and micelles, respectively, after being diluted to 40 μM. The H/D exchange is known to occur instantaneously when labile hydrogen atoms are exposed to D_2O without shielding of hydrogen bonding or hydrophobic chains[4]. Consistent with this principle, an instantaneous exchange is mainly observed in the charged lysine (K) region and partially in its adjacent β-sheet forming region of PA-1 polymers upon exposure to D_2O. This observation is indicated by the wide distribution of exchanged species (PA*n*D) that contain different number of exchanged deuterium atoms (*n*) ranging from 6 to 14 with the highest values at ϕ_{PA9D} and ϕ_{PA10D} (Fig. 4d). The exchange species of PA9D and PA10D are highly likely corresponding to the case where only the -NH and $-NH_2$ in charged region are fully exchanged with D_2O. With extended exposure time, more exchange of hydrogen atoms in the interior β-sheet forming region of PA-1 polymers occurs, as suggested by the increasing fractions of exchanged species with higher values of *n*. Importantly, there is no substantial increase in ϕ_{PA15D} and ϕ_{PA16D} within the entire measurement timespan of PA-1, whereas ϕ_{PA17D} rises significantly. This result suggests that the water diffusion into the three remaining hydrogen atoms of valine (V) is suppressed by the strong hydrogen bonding between monomers (Fig. 4g). Similarly, the increase in ϕ_{PA17D} is observed for PA-2 and PA-3 without substantial increases in ϕ_{PA15D} and ϕ_{PA16D}. Therefore, the H/D exchange resulting in the formation of PA17D species in our work is mainly contributed by the instant exposure of the entire monomer to D_2O, representing its extent of motion against the polymer backbone, rather than by the D_2O diffusion into interior polymers. Although the exact molecular pathway of this monomer motion remains elusive due to the limited temporal and spatial resolution associated with available experimental techniques, the overall exchange kinetics of fully exchange species (ϕ_{PA17D}) as a function of exposure time (*t*) can still serve as a highly effective indicator for the monomer motion against the supramolecular polymer backbones (Fig. 4h).

Figures

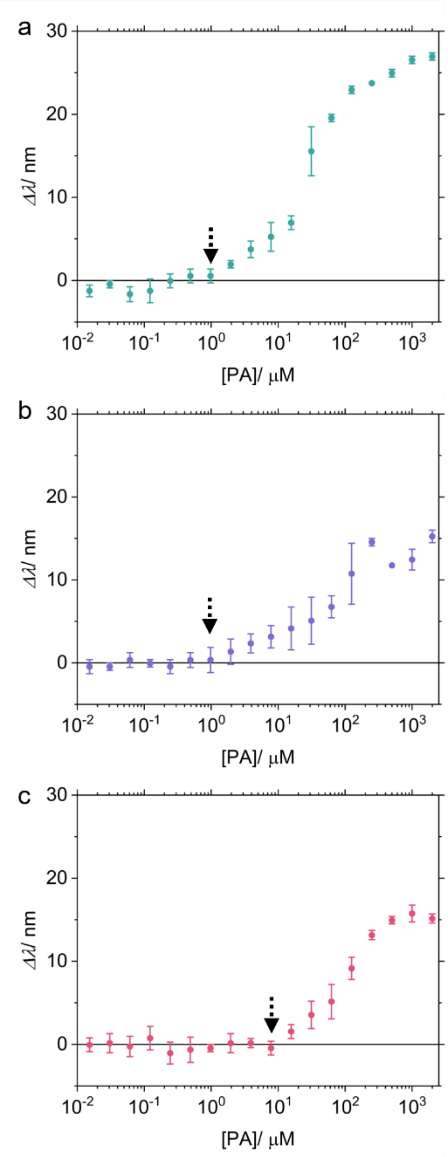

Supplementary Fig. 1. Nile Red assay of three PA samples prepared at [NaOH]/[PA] = 0. a-c, Blue shift ($\Delta\lambda$) of the emission spectra of Nile Red in **(a)** PA-1, **(b)** PA-2, and **(c)** PA-3 systems, relative to their counterpart in water at various [PA]. The critical micelle concentration (CMC) of these three PA molecules is around 1, 1, and 8 μM, respectively, as marked by the arrows. These results suggest that PA monomers primarily exist as micelles or polymers at the concentrations studied in the titrations and therefore the presence of free monomers can be negligible here. All measurements were taken from distinct samples (N = 5). Error bars denote the standard deviation (SD).

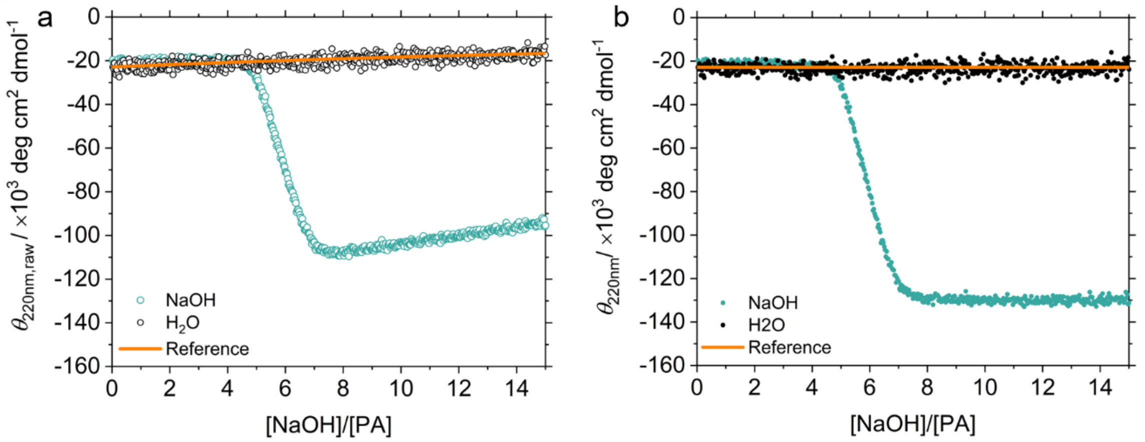

Supplementary Fig. 2. Correction of the dilution effect in the CD titration curves. a, Evolution of raw molar ellipticity at 220 nm ($\theta_{220\text{nm,raw}}$) measured when 0.75 ml of 800 µM NaOH solution is titrated to 2ml of 20 µM PA-1 ($C_{16}V_3A_3K_3$) solution. To test the dilution effect due to the addition of aqueous solution of NaOH, an equal volume of Milli-Q H_2O is also titrated to 2ml of 20 µM PA-1 solution. The reference line for the dilution effect is given by $\theta_{220\text{nm,raw}} = \theta_{220\text{nm,0}} \cdot V_0/(V_0 + V_{\text{H}_2\text{O or NaOH}})$, where $\theta_{220\text{nm,0}}$ and V_0 are the initial molar ellipticity and volume of PA-1 solution, respectively, and $V_{\text{H}_2\text{O or NaOH}}$ is the volume of titrated H_2O or NaOH. This line is consistent with the experimental data measured for the titration with water and for the titration with NaOH prior to the formation of β-sheet polymers. **b,** Evolution of molar ellipticity at 220 nm ($\theta_{220\text{nm}}$) after the correction of the dilution effect with $\theta_{220\text{nm}} = \theta_{220\text{nm,raw}}\,(V_0 + V_{\text{H}_2\text{O or NaOH}})/V_0$. The reference line is given by $\theta_{220\text{nm}} = \theta_{220\text{nm,0}}$, in good alignment with the corrected titration curves. These results indicate that the dilution effect here (maximum ×1.375 dilution) does not cause a noticeable influence on the polymerization of PA, and the above procedure can be applied to correct such effect. Therefore, the same correction procedure was applied for all the CD titration curves in this work, unless otherwise stated. To maintain the same dilution effect for the titrations done at different [PA], the concentration ratio of NaOH solution and PA solution was kept the same, as summarized in Supplementary Table 1.

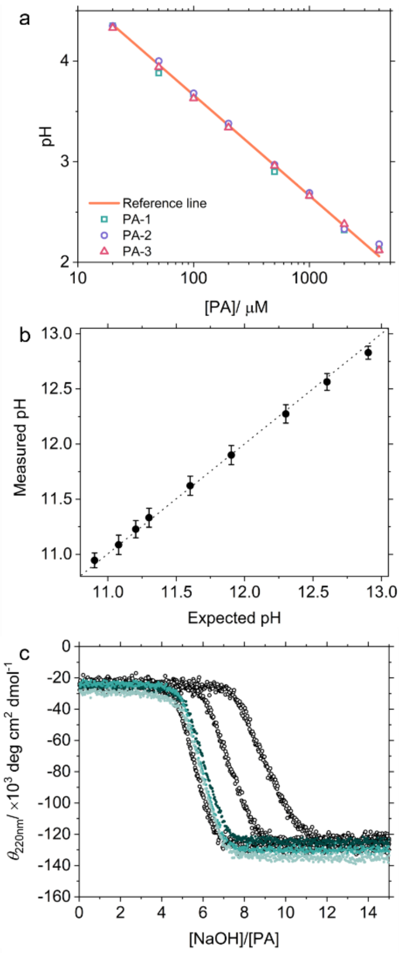

Supplementary Fig. 3. Essence for obtaining reproducible titration experiments. a, pH values of aqueous solutions containing three different PA molecules at various monomer concentrations. The reference line is the linear fit of the data, which gives: $pH = -\log_{10}[PA] + 5.66$ and $R^2 = 0.997$. The pH values of newly prepared stock solutions as well as their diluted solutions were always checked with respect to this reference line. **b,** pH values of NaOH solutions at various concentrations used for the titration experiments. The theoretically expected values are calculated with $pH_{\mathrm{NaOH}} = 14 + \log_{10}[NaOH]$. Similarly, the pH values of newly prepared NaOH solutions were always checked with respect to their theoretically expected values. Measurements were taken from distinct samples (N = 3). **c,** Representative CD titration curves of 20 µM PA-1 solutions using NaOH solution stored with different methods. When NaOH solution was stored in sealed 50 ml falcon tubes but was repeatedly taken for the titration experiments, the CD titration curves of PA-1 solutions of the same batch are not producible due to the continuous exposure of NaOH to atmospheric CO_2 during this process, as shown with the curves marked with empty symbols. To fix this issue, the freshly prepared stock solution of NaOH was always immediately split into 2 ml Eppendorf tubes and tightly sealed with Parafilm, which was only opened for usage right before the titration. This standard procedure enabled excellent reproducibility, as demonstrated with PA-1 solutions prepared from different batches throughout an experimental period of two years. Error bars denote the standard deviation (SD).

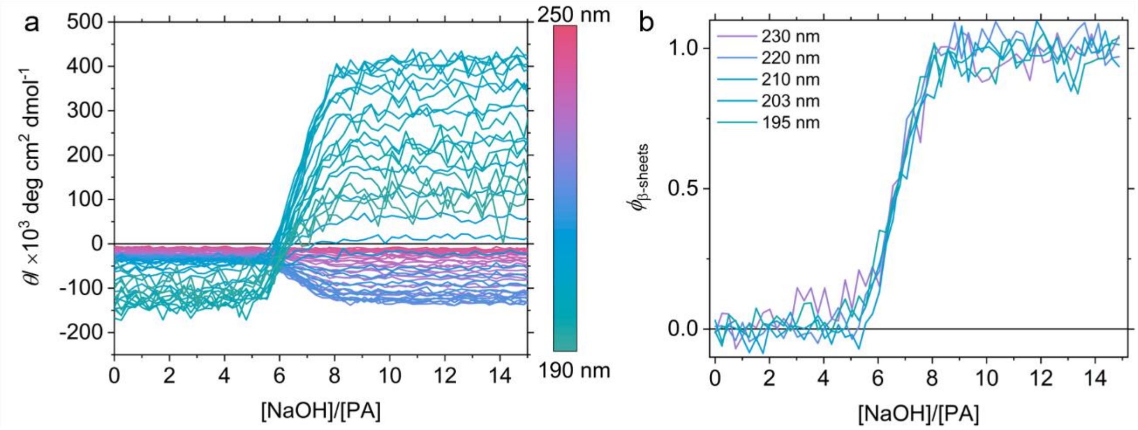

Supplementary Fig. 4. Wavelength dependence in the CD titration curves and their normalization. a, Molar ellipticity (θ) at different wavelengths (λ) as a function of the molar equivalents of titrated NaOH to PA monomers ([NaOH]/[PA]). **b,** Molar fraction of monomers within β-sheets ($\phi_{\beta-\mathrm{sheets}}$) as a function of [NaOH]/[PA], obtained by normalizing several representative curves in (A). The consistent normalization curves at different wavelengths suggest that the PA system can be well described by a linear combination of two species: random-coil micelles and β-sheet polymers. For the normalization process, we used the equation of $\phi_{\beta-\mathrm{sheets}} = (\theta - \theta_{\mathrm{initial\ random-coils}})/(\theta_{\mathrm{final\ \beta-sheets}} - \theta_{\mathrm{initial\ random-coils}})$. To minimize the influence of data noise, the data points in the [NaOH]/[PA] range of 0 to 1 were averaged to obtain $\theta_{\mathrm{initial\ random-coils}}$ and those in the range of 14 to 15 were averaged to obtain $\theta_{\mathrm{final\ \beta-sheets}}$ for all the CD titration curves unless otherwise stated.

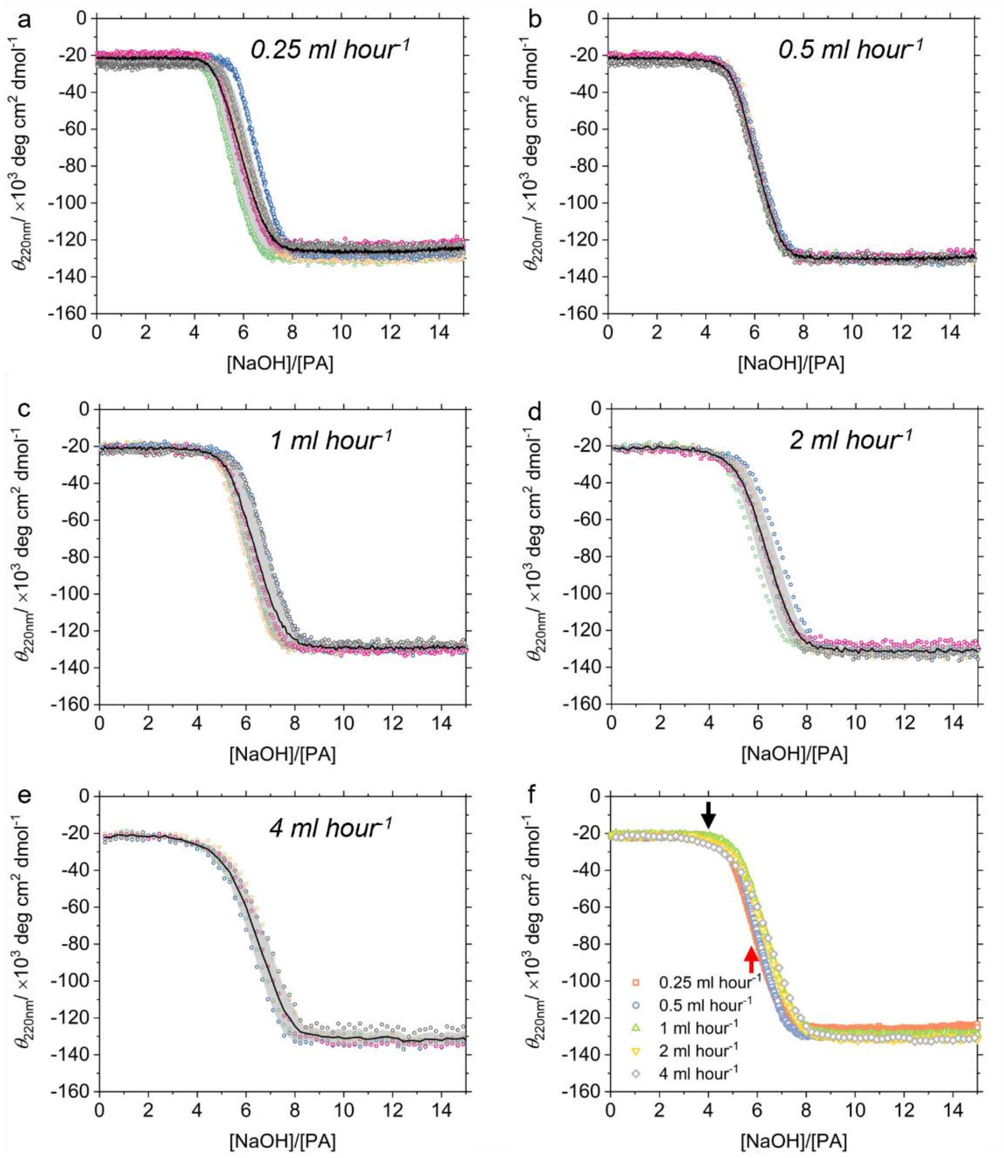

Supplementary Fig. 5. Dependence of the CD titration curves on the titration rates. a-e, $\theta_{220\mathrm{nm}}$ measured for the titrations of 800 μM NaOH solution to 20 μM PA-1 solution conducted at various titration rates, ranging from 0.25 to 4 ml hour^{-1}. Five repetitions at each rate, their average values and corresponding SD values are shown with symbols, lines and shadows, respectively. **f,** Comparison of the average CD titration curves obtained at different titration rates. These CD titration curves display a dependence on the titration rate. When the titration rate is sufficiently low, such as at 0.5 ml hour^{-1} or lower, the titration curves overlap, indicating the formation of PA polymers under thermodynamic equilibrium conditions. All measurements were taken from distinct samples (N = 5). Error bars denote the standard deviation (SD).

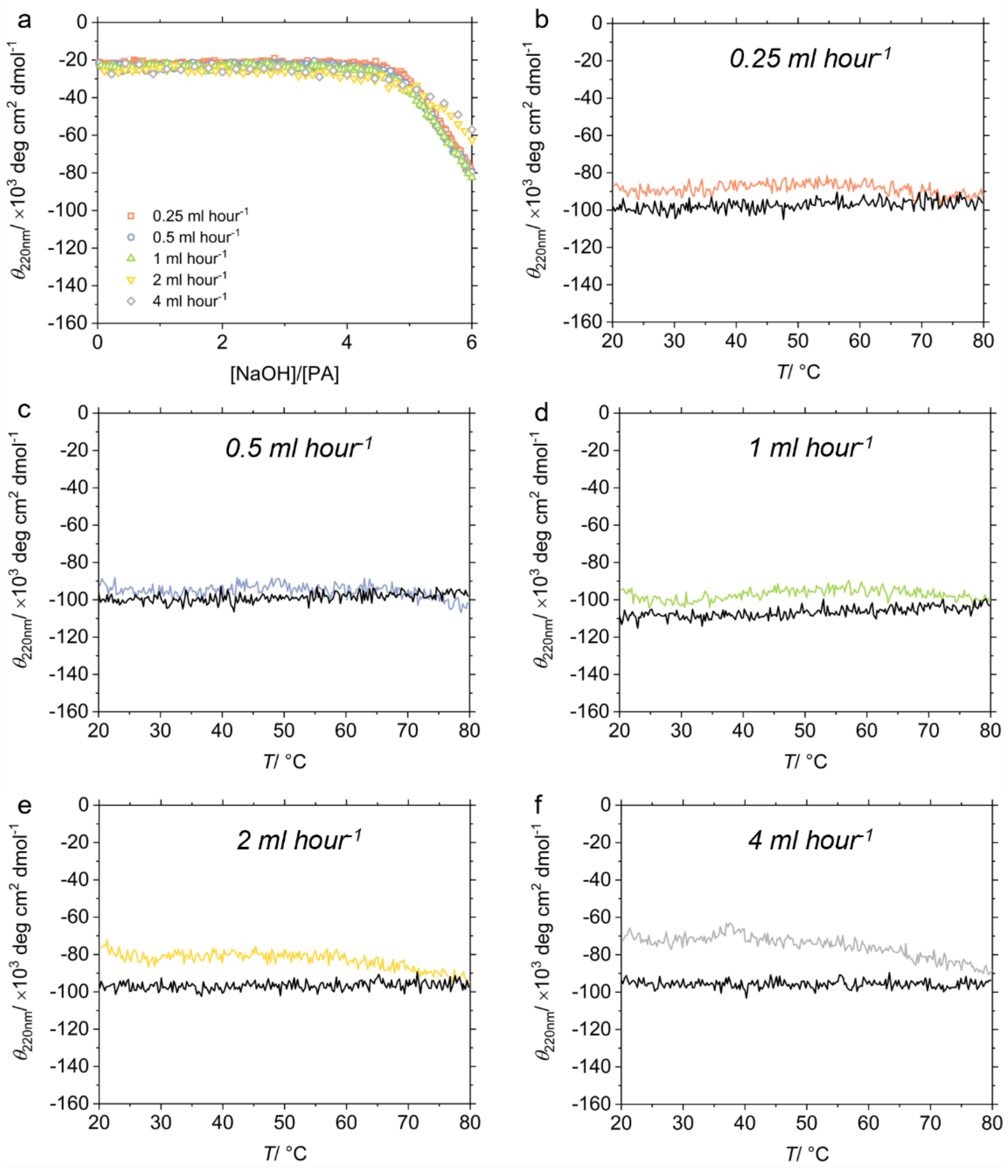

Supplementary Fig. 6. Annealing experiments to verify the formation of PA polymers under thermodynamic equilibrium conditions. a, $\theta_{220\text{nm}}$ measured for the titrations of 800 μM NaOH solution to 20 μM PA-1 solution conducted at various titration rates, ranging from 0.25 to 4 ml hour^{-1}. All these titrations are stopped at [NaOH]/[PA] = 6, followed by a heating and cooling procedure. **b-f,** $\theta_{220\text{nm}}$ as a function of the sample temperature (*T*), monitored during the heating (colorful lines) and cooling (black lines) process. Both the heating and cooling rates are 1 °C min^{-1}. All the samples were also annealed at 80 °C for 30 minutes. Both the CD titration and annealing curves show a strong dependence on the titration rate. Only when the titration rate is sufficiently low, like at 1 ml hour^{-1} or lower, do the titration curves become overlapping and the molar ellipticities remain the same before and after the heating-cooling process, thereby enabling the formation of PA polymers under thermodynamic equilibrium conditions. To ensure the formation of PA polymers under such equilibrium conditions, we chose 0.5 ml hour^{-1} for all the titration experiments.

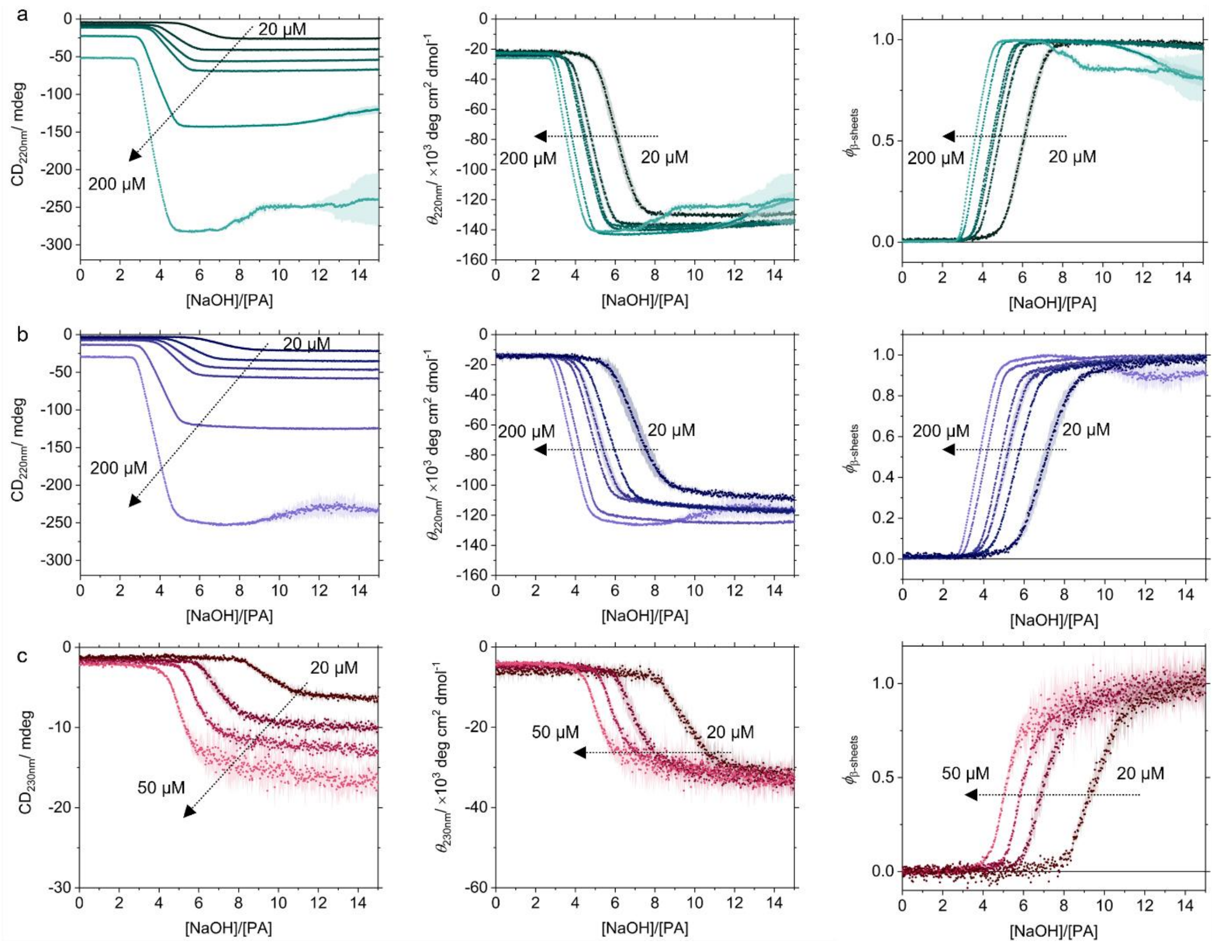

Supplementary Fig. 7. *In-situ* CD titration curves of three PA homopolymerization systems at different [PA]. a-b, Raw CD signals measured at λ = 220 nm ($CD_{220\text{nm}}$), the corresponding molar ellipticity ($\theta_{220\text{nm}}$), and $\phi_{\beta-\text{sheets}}$ obtained after the normalization as a function of [NaOH]/[PA] in **(a)** PA-1 and **(b)** PA-2 ($C_{16}V_3G_3K_3$) systems. In the direction of arrows, [PA] increases in the order of 20 μM, 30 μM, 40 μM, 50 μM, 100 μM and 200 μM. **c,** Same set of CD titration results measured at the wavelength of λ = 230 nm for PA-3 (($C_{16}A_3G_3K_3$)) system, due to the linear dichroism issue as detailed in Supplementary Fig. 8. In the direction of arrows, [PA] increases in the order of 20 μM, 30 μM, 40 μM, and 50 μM. For PA-1 at [PA] = 100 μM and 200 μM as well PA-2 at [PA] = 200 μM, the fluctuation in CD intensity observed at the end of titration is caused by the scattering of concentrated PA polymers under the stirring conditions. As this phenomenon only occurs after the appearance of the second plateau corresponding to the completion of β-sheet polymer formation, its influence on the equilibrium between micelles and polymers can be neglected. Hence, the normalization process was performed in the region prior to the occurrence of scattering events for these curves. All measurements were taken from distinct samples (N = 5). Error bars denote the standard deviation (SD).

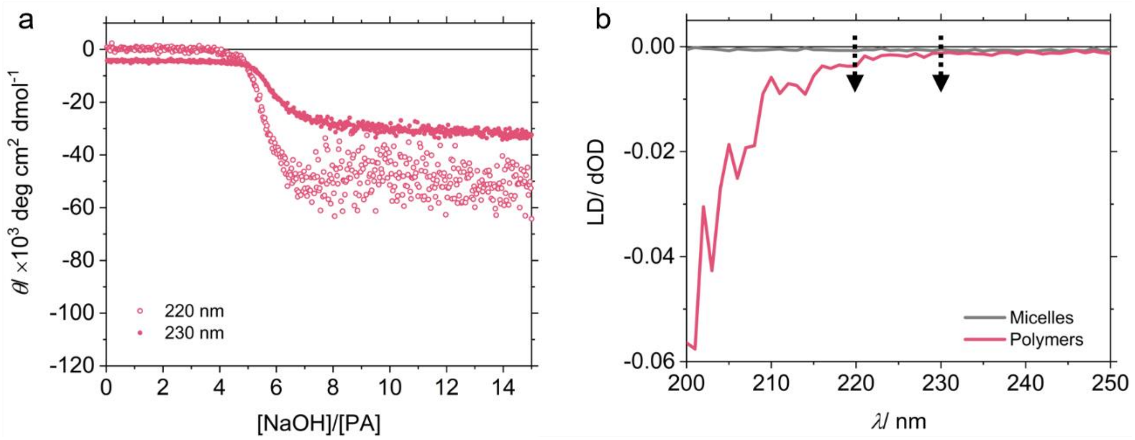

Supplementary Fig. 8. Linear dichroism (LD) issue in the *in-situ* titrations of PA-3 system. **a,** *In-situ* CD titration curves of PA-3 at [PA] = 40 μM measured at different wavelengths (λ). In contrast to the curve measured at λ = 230 nm, the one measured at λ = 220 nm fluctuates highly in the [NaOH]/[PA] range from 7 to 15. **b,** Linear dichroism signal of PA-3 micelles formed at [NaOH]/[PA] = 0 (before the titration) and polymers formed at [NaOH]/[PA] = 15 (after the titration). The micellar sample shows a negligible LD signal. However, the PA-3 polymer sample displays a substantial LD signal at low wavelengths, such as 220 nm, possibly due to the linear alignment of long PA-3 polymers under stirring [5]. This effect can generate artifact CD signals and thereby lead to fluctuations in **(a)**. Importantly, the LD signal of PA-3 polymers at λ = 230 nm is as small as that of micelles. This observation provides the possibility of measuring reliable *in-situ* titration curves of PA-3 systems up to 50 μM, as demonstrated in Supplementary Fig. 7c.

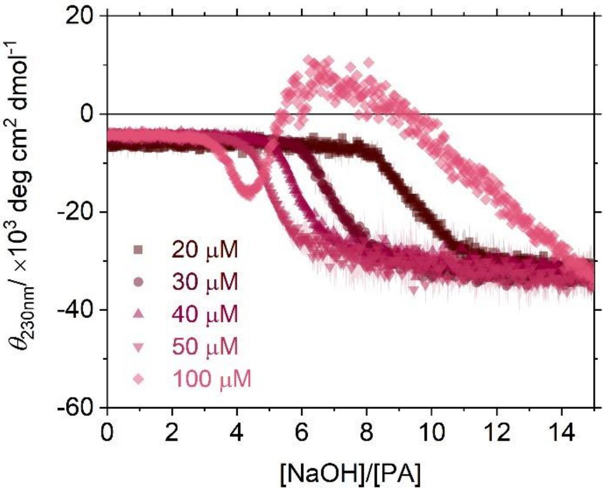

Supplementary Fig. 9. ***In-situ*** **CD titration curves of PA-3 at higher concentrations.** When the [PA] increased to 100 μM for the PA-3 system, a non-negligible LD effect is present, which substantially distorts the titration curves. Nevertheless, the consistent molar ellipticity of PA-3 polymers formed at lower [PA] suggest a negligible influence of LD on their CD signals. All measurements were taken from distinct samples (N = 5). Error bars denote the standard deviation (SD).

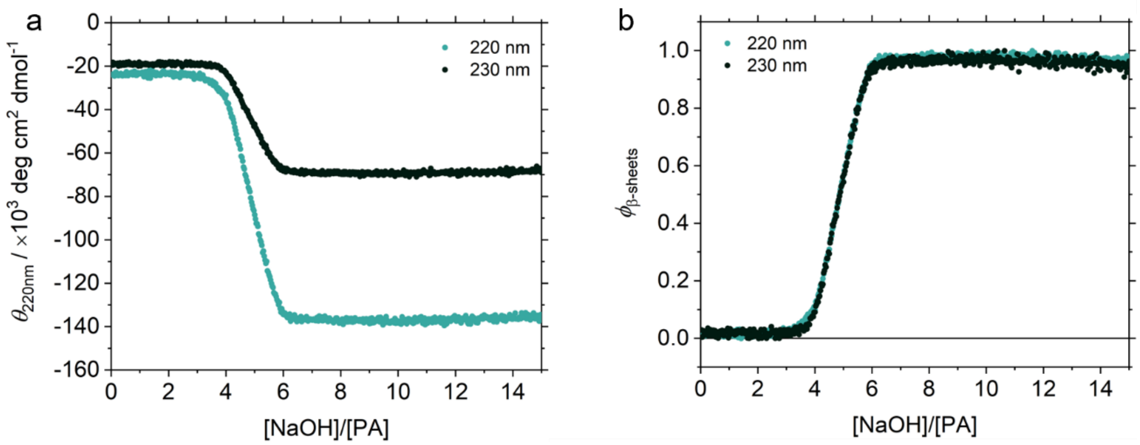

Supplementary Fig. 10. Wavelength dependence in the CD titration curves and their normalization. a, CD titration curves measured at two different wavelengths (λ): 220 nm and 230 nm. **b,** The corresponding normalized curves. In good alignment with Supplementary Fig. 4, the consistent normalized titration curves obtained at these two wavelengths suggest that PA system can be approximated as a binary system composed of random-coil micelles and β-sheet polymers. Furthermore, these results indicate that the CD titration curves obtained at different wavelengths in this work should equally represent the formation process of PA polymers, when the LD signal is not significant.

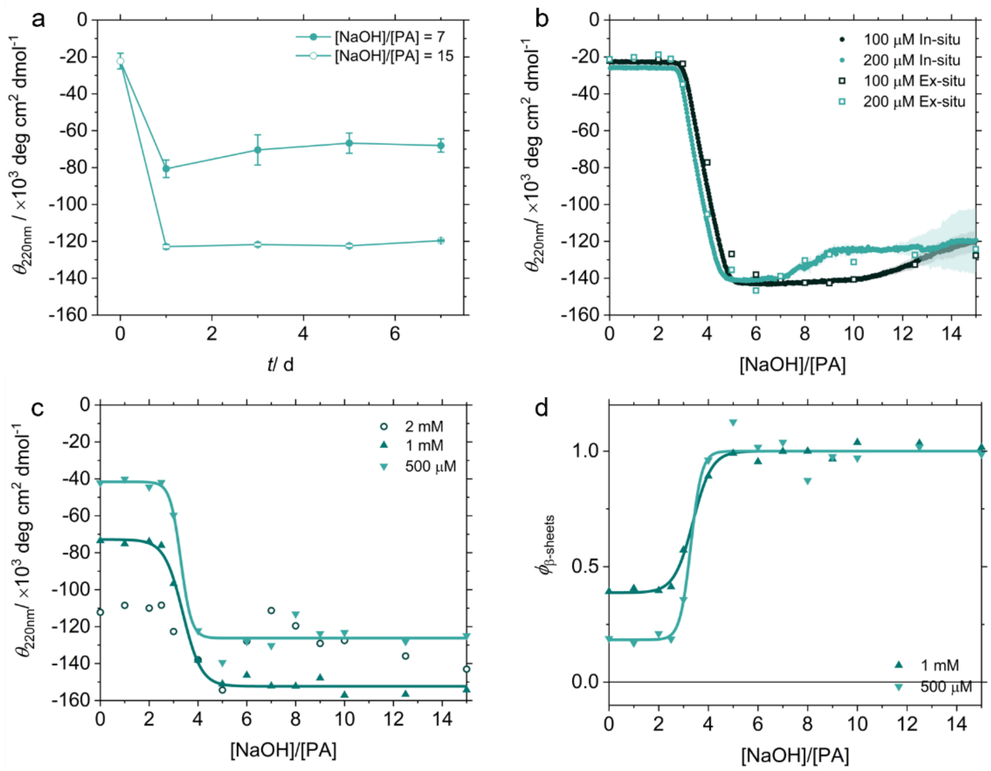

Supplementary Fig. 11. *Ex-situ* CD titration curves of PA-1. a, Polymerization kinetics of PA-1 polymers prepared using the *ex-situ* titration method at [PA] = 20 μM and [NaOH]/[PA] = 7 and 15. The CD signal of the polymers appears to be consistent after 3 days, indicating that it takes approximately 3 days for the polymers to reach thermodynamic equilibrium states. To ensure that all the PA samples prepared by the *ex-situ* titration method reached equilibrium, they were always aged at room temperature for 7 days prior to any further characterizations. Measurements were taken from distinct samples (N = 3). Error bars denote the standard deviation (SD). **b,** Comparison of the CD titration curves obtained using *in-situ* and *ex-situ* titration methods. The *in-situ* CD data is of higher quality and throughput than the *ex-situ* ones, yet both sets of data still qualitatively agree with each other. Hence, for the PA samples with higher [PA] ranging from 500 μM to 4 mM, they were prepared by the *ex-situ* method. This was necessary since PA samples at such high concentrations in a 10 mm light path cuvette can cause saturated HT in the CD measurement [6]. However, the commercially available cuvettes with the much smaller light path cannot allow for stirring during the *in-situ* titration. **c**, *Ex-situ* CD titration curves and **d,** their normalization of PA-1 at higher [PA]. The absolute values of molar ellipticity (θ) for these samples at [NaOH]/[PA] =0 are significantly higher than those of random-coil micelles in the [PA] range from 20 μM to 200 μM, as shown in Supplementary Fig. 7 and 24. Thus, for the normalization process, we used the equation $\phi_{\beta-\text{sheets}} = (\theta - \theta_{\text{initial random-coils}})/(\theta_{\text{final }\beta-\text{sheets}} - \theta_{\text{initial random-coils}})$, where $\theta_{\text{initial random-coils}}$ was taken from the average values of PA-1 random-coil micelles with [PA] from 20 μM to 200 μM. For [PA] = 2 mM, the CD signals are highly fluctuated due to the pronounced LD artifact associated with the highly concentrated polymers in the aqueous solution. Consequently, the normalization process was not applied to this curve. In **(c)** and **(d)**, reference lines are given by fitting the titration curves using the Boltzmann function, as detailed in Supplementary Table 2, to better illustrate the trend of polymerization.

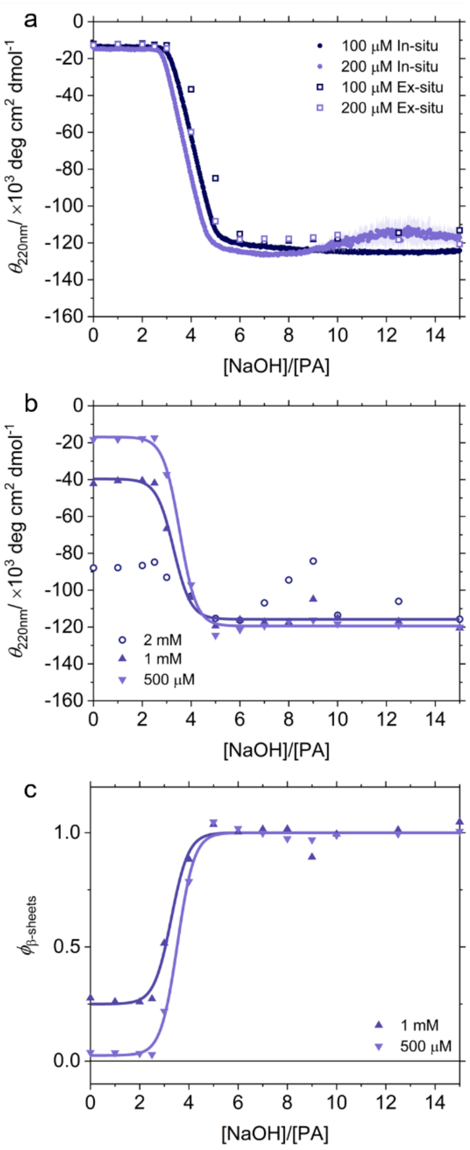

Supplementary Fig. 12. *Ex-situ* CD titration curves of PA-2. a, Comparison of the CD titration curves obtained using *in-situ* and *ex-situ* titration methods. **b-c,** *Ex-situ* CD titration curves **(b)** and their normalization at higher values of [PA] **(c)**. Similarly, $\theta_{\mathrm{initial\ random-coils}}$ was taken from the average values of PA-2 random-coil micelles with [PA] from 20 μM to 200 μM during the normalization process. For [PA] = 2 mM, the CD titration curve was not normalized due to the presence of pronounced LD artifact. In **(b)** and **(c)**, reference lines are given by fitting the titration curves using the Boltzmann function, as detailed in Supplementary Table 3, to better illustrate the trend of polymerization. Error bars denote the standard deviation (SD).

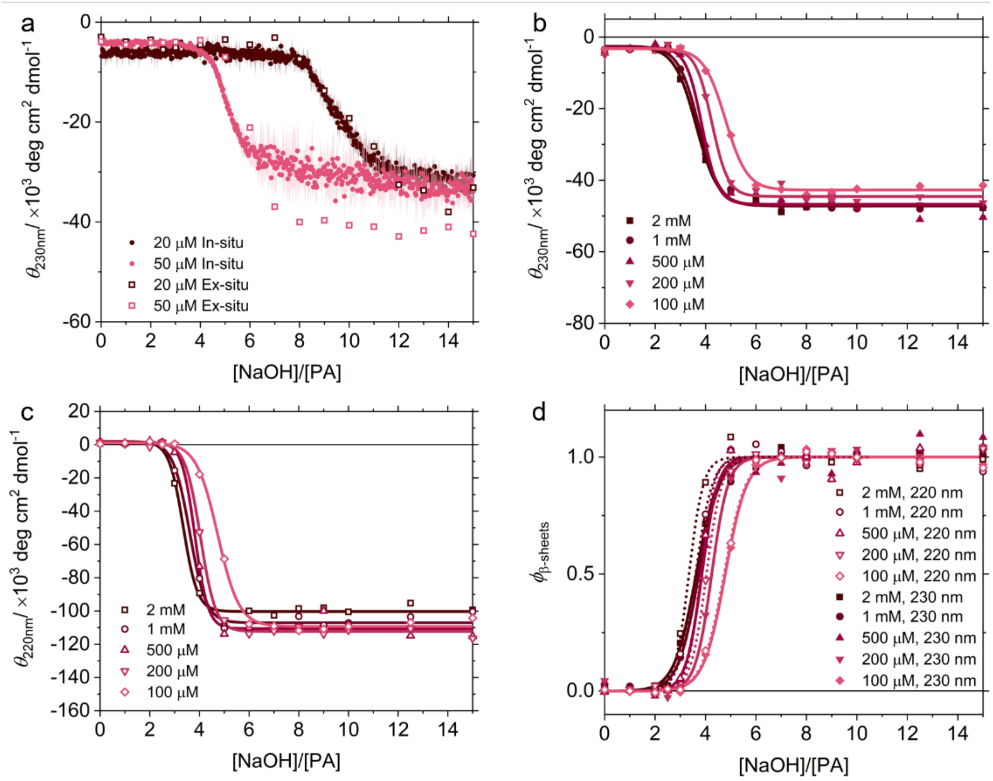

Supplementary Fig. 13. *Ex-situ* CD titration curves of PA-3. a, Comparison of the CD titration curves obtained using *in-situ* and *ex-situ* titration methods. **b-c,** *Ex-situ* CD titration curves at higher values of [PA] measured at **(b)** λ = 230 nm and **(c)** λ = 220 nm. **d,** Normalized titration curves measured at two different wavelengths. The values of $\theta_{\mathrm{initial\ random-coils}}$ are consistent for all PA-3 samples at different [PA], indicating that PA-3 samples formed at [NaOH]/[PA] = 0 are micelles in this range of concentration. In **(b)** to **(d)**, reference lines are determined by fitting the titration curves using the Boltzmann function, as detailed in Supplementary Table 4 and 5, to better illustrate the trend of polymerization. Error bars denote the standard deviation (SD).

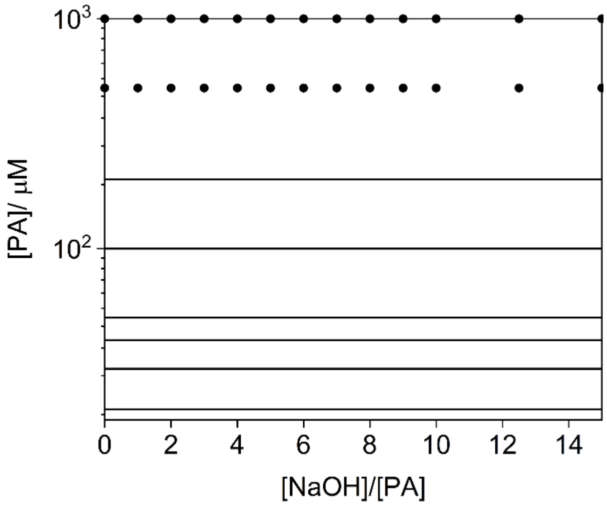

Supplementary Fig. 14. Experimental data points used in the construction of the assembly landscape. For the PA-1 and PA-2 systems, data points were acquired using *in-situ* CD titration at the monomer PA concentrations ([PA]) ranging from 20 to 200 μM. In contrast, data points were obtained through *ex-situ* CD titration at [PA] of 500 and 1000 μM. In the case of the PA-3 system, data points at [PA] of 100 and 200 μM were obtained using *ex-situ* CD titration method instead of *in-situ* one due to the non-negligible LD effect at these high concentrations.

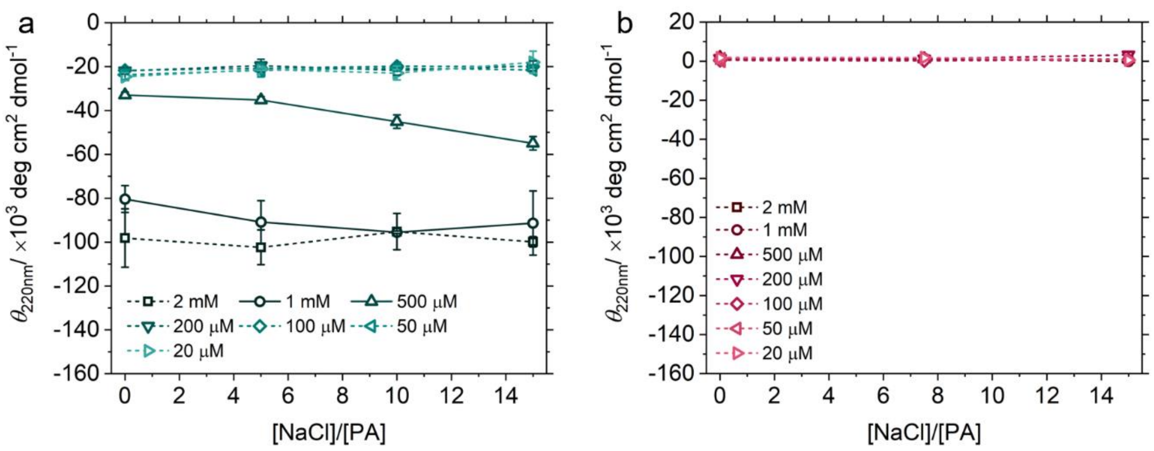

Supplementary Fig. 15. *Ex-situ* CD titration curves of (a)PA-1 and (b) PA-3 with NaCl as titrant control for the salt generated during NaOH titration. For PA-1 in (a), at monomer concentrations of 20–200 μM or 2 mM, the effect of NaCl is negligible, as indicated by the nearly constant molar ellipticity throughout the titration. However, at intermediate concentrations (e.g., 500 μM and 1 mM), NaCl induces a slight increase in absolute molar ellipticity values, suggesting a minor enhancement in the fraction of polymers. Nevertheless, compared to that of NaOH shown in Supplementary Fig. 11, this effect remains insignificant. In contrast, for PA-3 in (b), NaCl does not initiate polymerization at any concentration. Thus, the charge screening effect of NaCl generated during NaOH titration can be reasonably neglected in our analysis. Measurements in (a) were taken from distinct samples (N = 3). Error bars denote the standard deviation (SD).

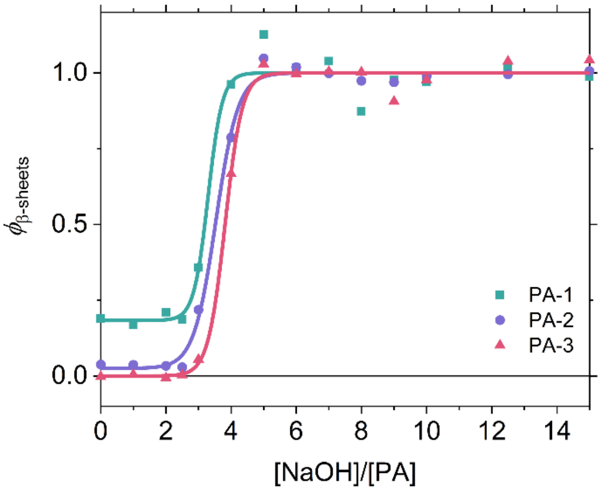

Supplementary Fig. 16. Slice of the assembly landscape at [PA] = 500 μM. $\phi_{\beta-\mathrm{sheets}}$ in three PA homopolymerization systems is plotted as a function of [NaOH]/[PA]. The experimentally obtained data are marked by symbols and the Boltzman fits of these data are illustrated by lines.

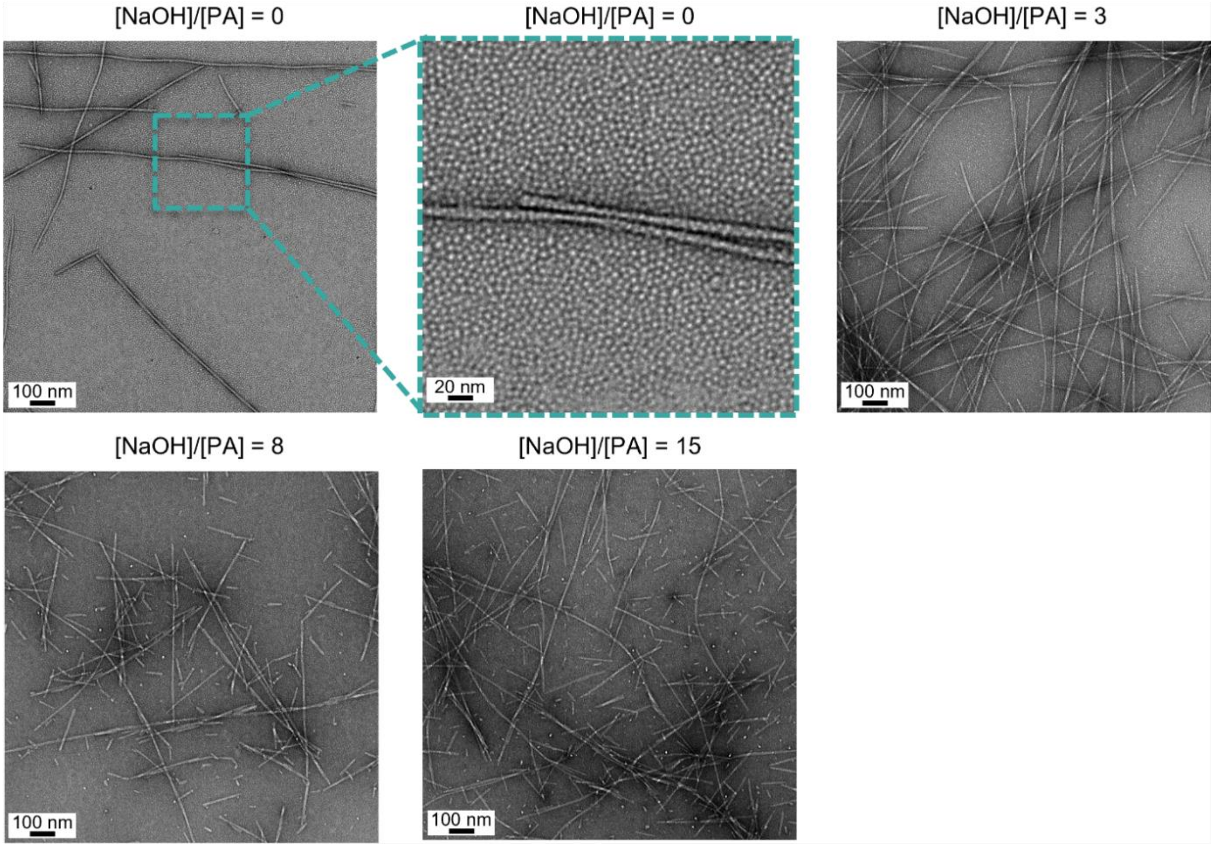

Supplementary Fig. 17. Representative negative-staining TEM images of the PA-1 homopolymerization system at different [NaOH]/[PA] with [PA] = 500 μM. At [NaOH]/[PA] = 0 and therefore $\phi_{\beta-\text{sheets}} \approx 0.2$, numerous micelles coexist with the polymers.

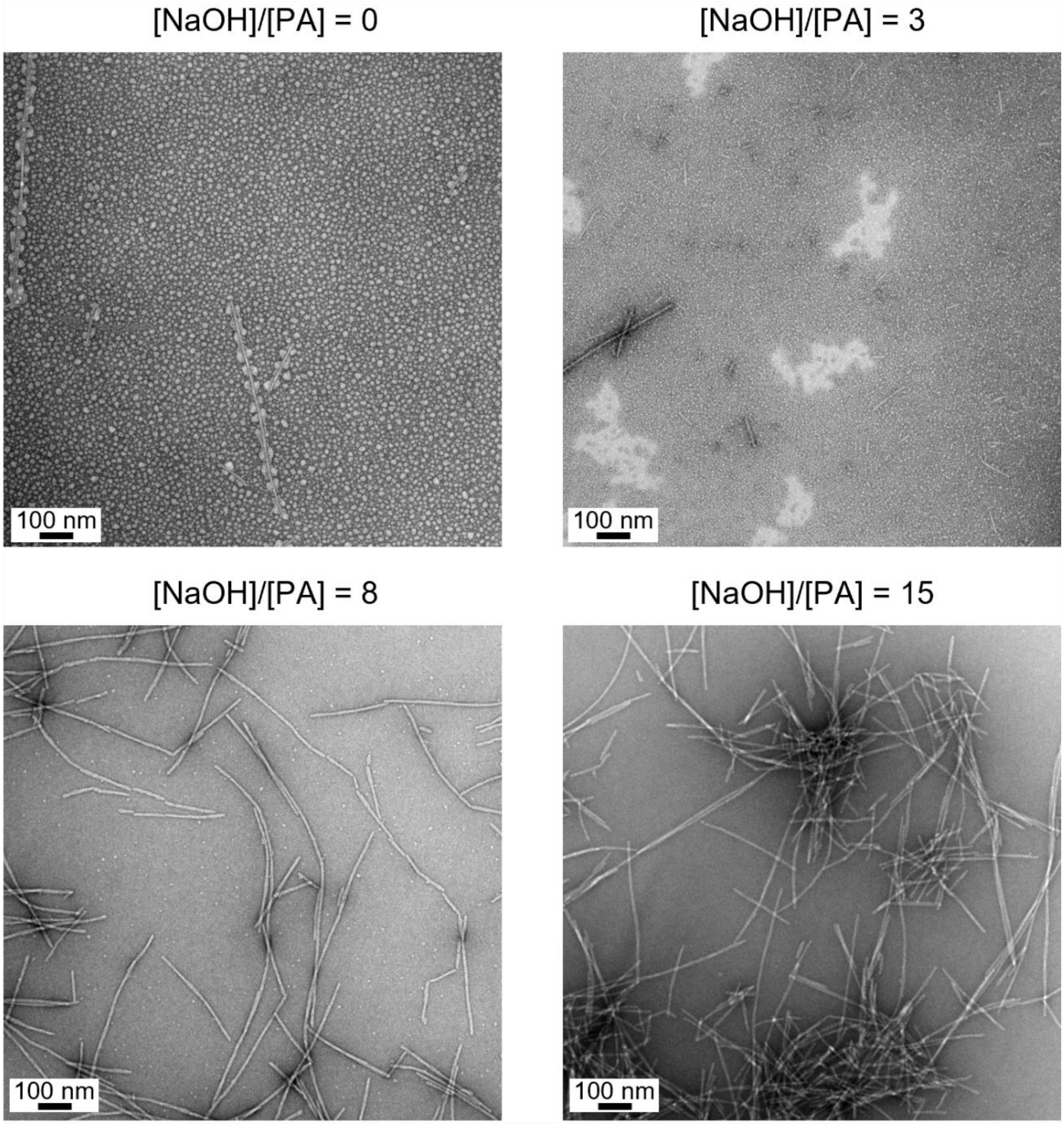

Supplementary Fig. 18. Representative negative-staining TEM images of the PA-2 homopolymerization system at different [NaOH]/[PA] ratios with [PA] = 500 µM. At [NaOH]/[PA] = 0 and therefore $\phi_{\mathrm{\beta-sheets}} \approx 0.03$, numerous micelles coexist with a few polymers.

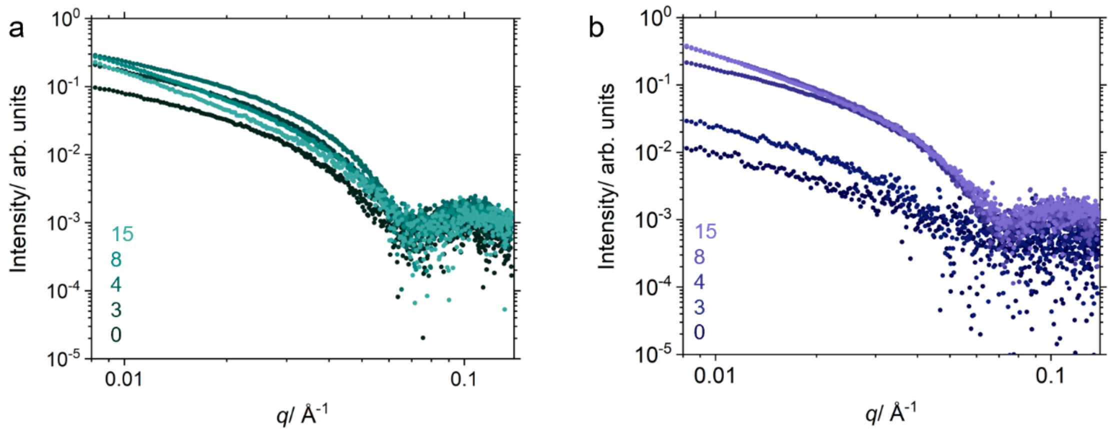

Supplementary Fig. 19. Representative SAXS profiles of PA homopolymerization systems at different [NaOH]/[PA] ratios: (a) PA-1 and (b) PA-2. [PA] was fixed at 500 µM for both systems. When a substantial fraction of micelles coexists with polymers, for example in PA-2 at [NaOH]/[PA] = 0 ($\phi_{\beta-\text{sheets}} \approx 0.03$), the scattering intensity of resulting mixture is significantly smaller than that of polymer samples with $\phi_{\beta-\text{sheets}} \approx 1$ formed at [NaOH]/[PA] = 15.

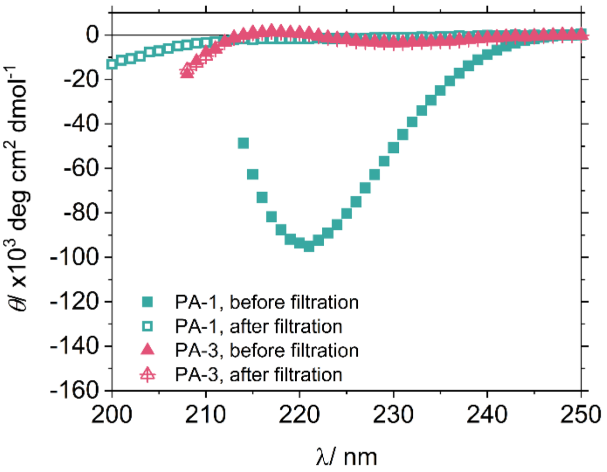

Supplementary Fig. 20. Indirect proof of the distinct sizes of PA micelles and polymers. CD spectra of PA-1 and PA-3 samples formed at [NaOH]/[PA] = 0 and [PA] = 2 mM before and after the filtration through a polyvinylidene fluoride (PVDF) syringe filter with an average pore size of 220 nm. Under these conditions, the formed PA-1 sample is mainly composed of polymers, whereas the PA-3 sample consists solely of micelles, as shown in Supplementary Fig. 11 and 13. For the PA-3 micelle sample, the nearly identical random-coil CD patterns before and after filtration suggest that the size of all micelles is smaller than 220 nm, which enables them to pass through the nanopores of the filter. By contrast, the disappearance of the β-sheet CD pattern and emergence of random-coil CD pattern in PA-1 sample upon filtration indicates that most PA polymers are too large to pass through the filter, with only a small fraction of random-coil micelles being sufficiently small to penetrate the filter. This observation is consistent with TEM results. In the CD spectra, only reliable data points with HT values lower than 700 V are plotted [6].

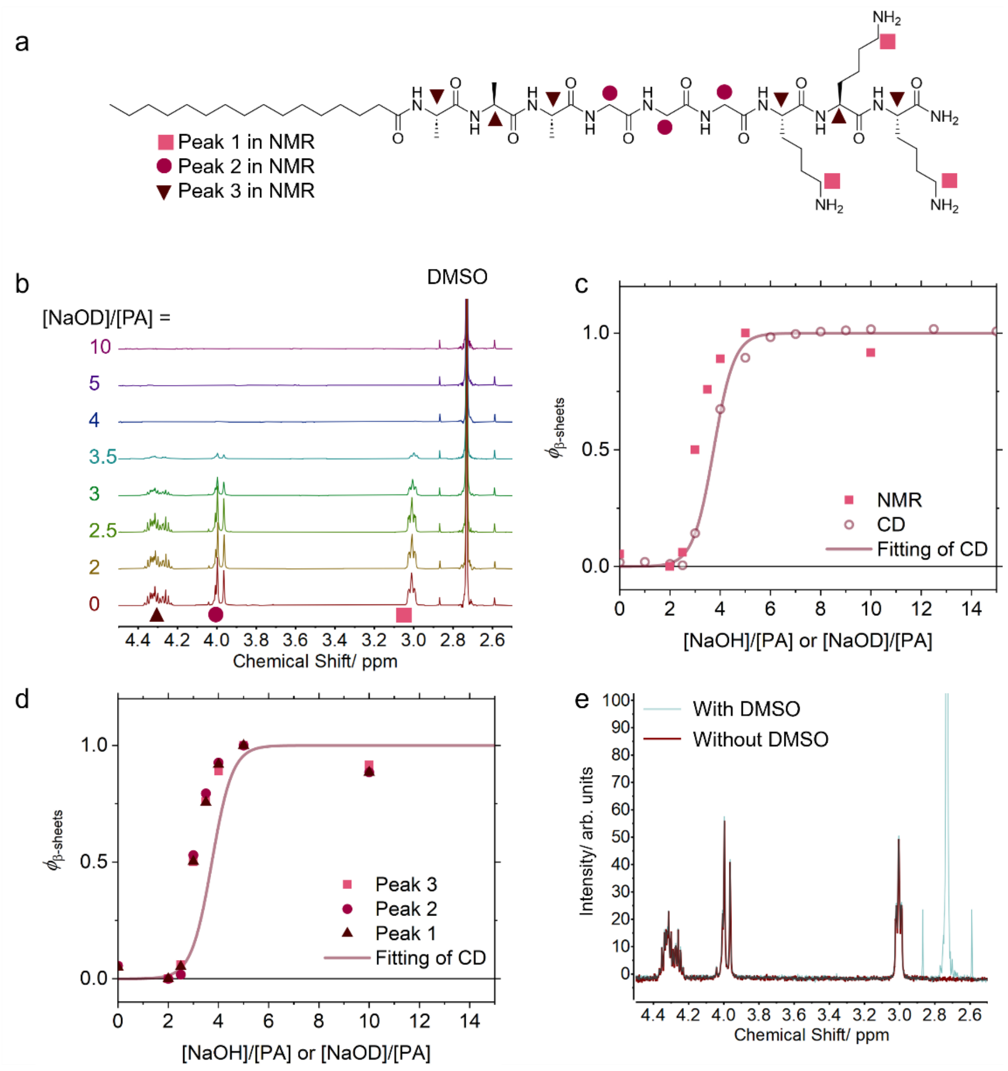

Supplementary Fig. 21. ^{1}H NMR characterization of PA-3 titrated with different equivalents of NaOD at [PA] = 1 mM. (a) Chemical structure of PA-3 with labeled protons corresponding to peaks in (b). (b) ^{1}H NMR spectra of PA-3 with different [NaOD]/[PA] ratios, demonstrating PA micelles are NMR-visible while polymers are NMR-silent. These results suggest that the negligible presence of micelles in the second region of CD titration curves. (c) NMR titration curves: the molar fraction of monomers within β-sheets ($\phi_{\beta-\text{sheets}}$) as a function of [NaOD]/[PA], obtained by normalizing the integration of NMR peak 1. The DMSO peak is used as an internal reference to calibrate integration values. CD titration curve and its fit at the same concentration are provided for comparison. (d) comparison of NMR titration curves extracted from the integration of different peaks in (a). (e) Comparison of the NMR spectra at [NaOD]/[PA] = 3, with and without DMSO as the internal reference in the D_2O. The nearly identical NMR signals corresponding to protons in PA confirm the negligible influence of DMSO on the assembly equilibrium between micelles and polymers.

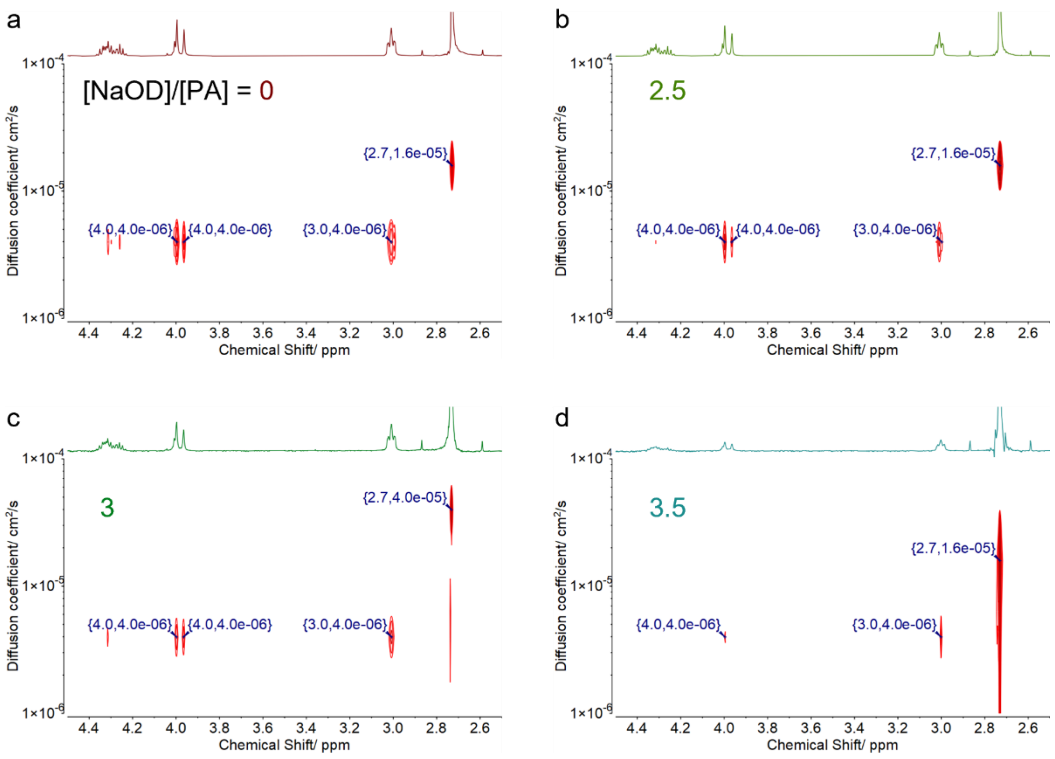

Supplementary Fig. 22. 2D DOSY NMR spectra of PA-3 titrated with different [NaOD]/[PA] ratios: (a) 0, (b) 2.5, (c) 3, and (d) 3.5. The diffusion coefficient (D) of PA micelles remains constant at 4.0×10^{-6} cm^2 s^{-1} across all samples, despite different fractions of micelles. This result indicates that the micelles maintain a consistent size throughout the titration process, supporting the binary micelle-polymer nature of our PA systems.

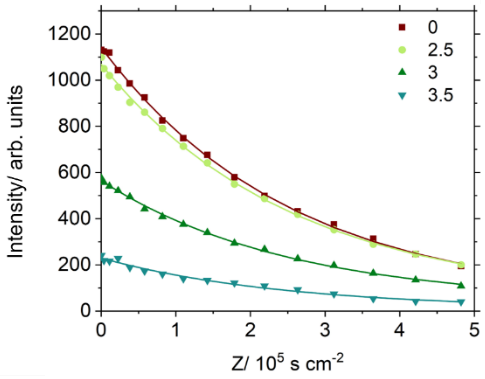

Supplementary Fig. 23. Extraction of diffusion coefficients (D) through fits of the integration of Peak 1 in the DOSY experiments. Peak 1 corresponds to the protons of the methylene groups adjacent to the amine group in the lysine residues. Fits with the model $y = a + b \cdot e^{-x \cdot D}$ are indicated in sold lines with experimental data shown as symbols. The extracted values of D are 3.95 × 10^{-6} cm^2 s^{-1}, 3.90 × 10^{-6} cm^2 s^{-1}, 3.98 × 10^{-6} cm^2 s^{-1}, and 4.07 × 10^{-6} cm^2 s^{-1} for [NaOD]/[PA] = 0, 2.5, 3, and 3.5, respectively. These consistent diffusion coefficients indicate uniform micelle size throughout the titration experiments.

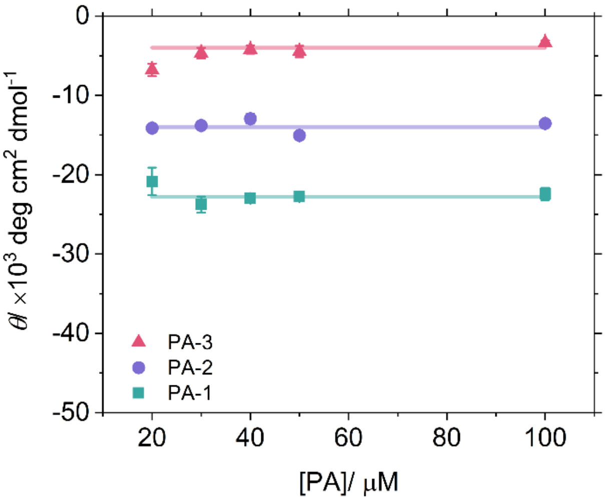

Supplementary Fig. 24. Presence of free monomers in PA systems. $\theta_{220\mathrm{nm}}$ measured for PA-1 as well as PA-2 and $\theta_{230\mathrm{nm}}$ measured for PA-3 are plotted against total monomer concentration ([PA]). [NaOH]/[[PA] was kept at 0. Linear fits of these data with a fixed slope of 0 are indicated by the lines. No PA polymers are expected to form under these conditions, as demonstrated in Supplementary Fig. 11 to 13. Free monomers are expected to exhibit a distinct molar ellipticity (θ) compared to micelles. Therefore, a detectable presence of free monomers in PA systems would be expected to decrease the absolute values of θ with the decreasing total monomer concentration ([PA]). However, the values of θ in all three PA systems are nearly constant when [PA] is varied between 20 μM and 100 μM, indicating a negligible presence of free monomers here. All measurements were taken from distinct samples (N = 3). Error bars denote the standard deviation (SD).

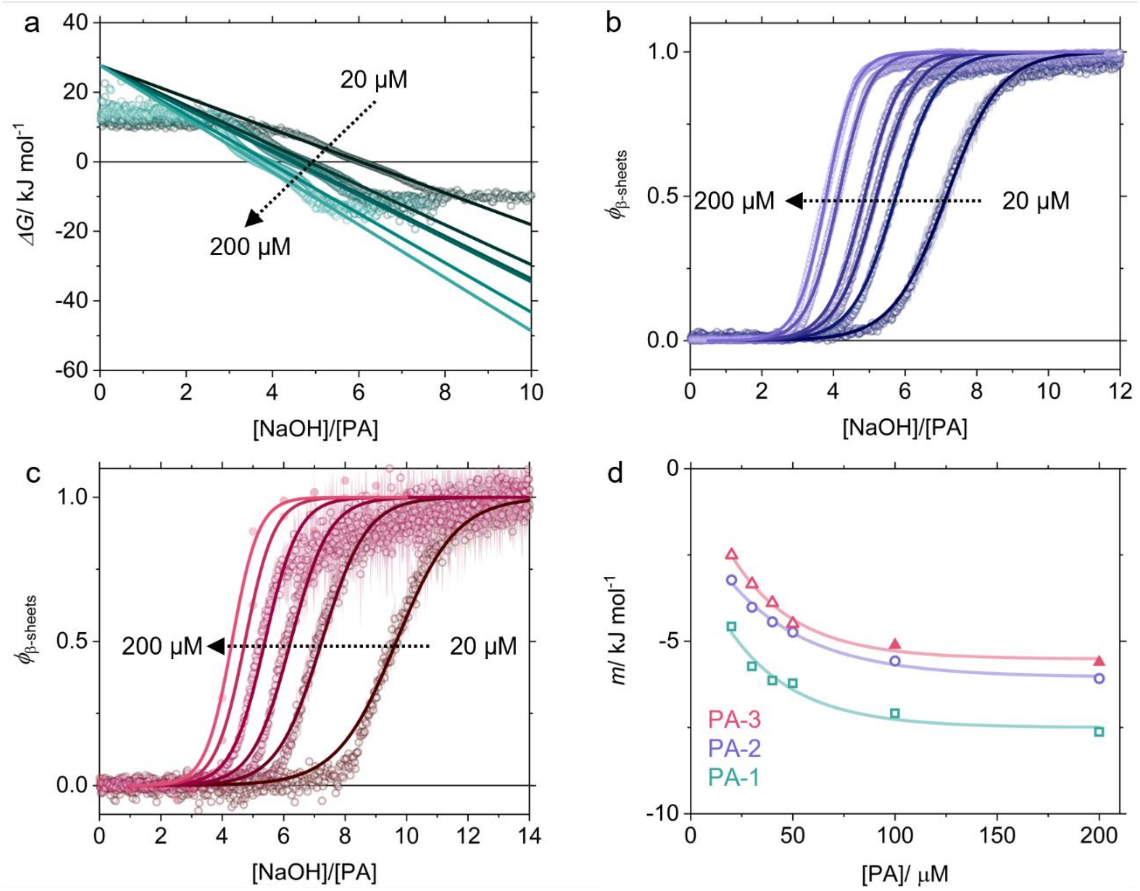

Supplementary Fig. 25. ***m*-factor mass balance model. a,** Fits (solid lines) of experimentally obtained ΔG curves (circles) of PA-1 at different [PA] using the *m*-factor model. **b-c,** Comparison of experimentally obtained normalized CD titration curves (circles) and fits (solid lines) for **(b)** PA-2 and **(c)** PA-3. **d,** Comparison of *m*-factor values obtained from the fits. The reference lines are provided by fitting the data with an exponential decay function $m = m_0 + A \cdot e^{-k([PA]-[PA]_0)}$) to better illustrate their trend against [PA]. For [PA] = 100 µM and 200 µM in the PA-3 system, the experimental CD titration curves used for fitting were obtained using the *ex-situ* titration method due to the LD issue for the *in-situ* titration method at these high concentrations as previously discussed. Measurements were taken from distinct samples (N = 5). Error bars denote the standard deviation (SD).

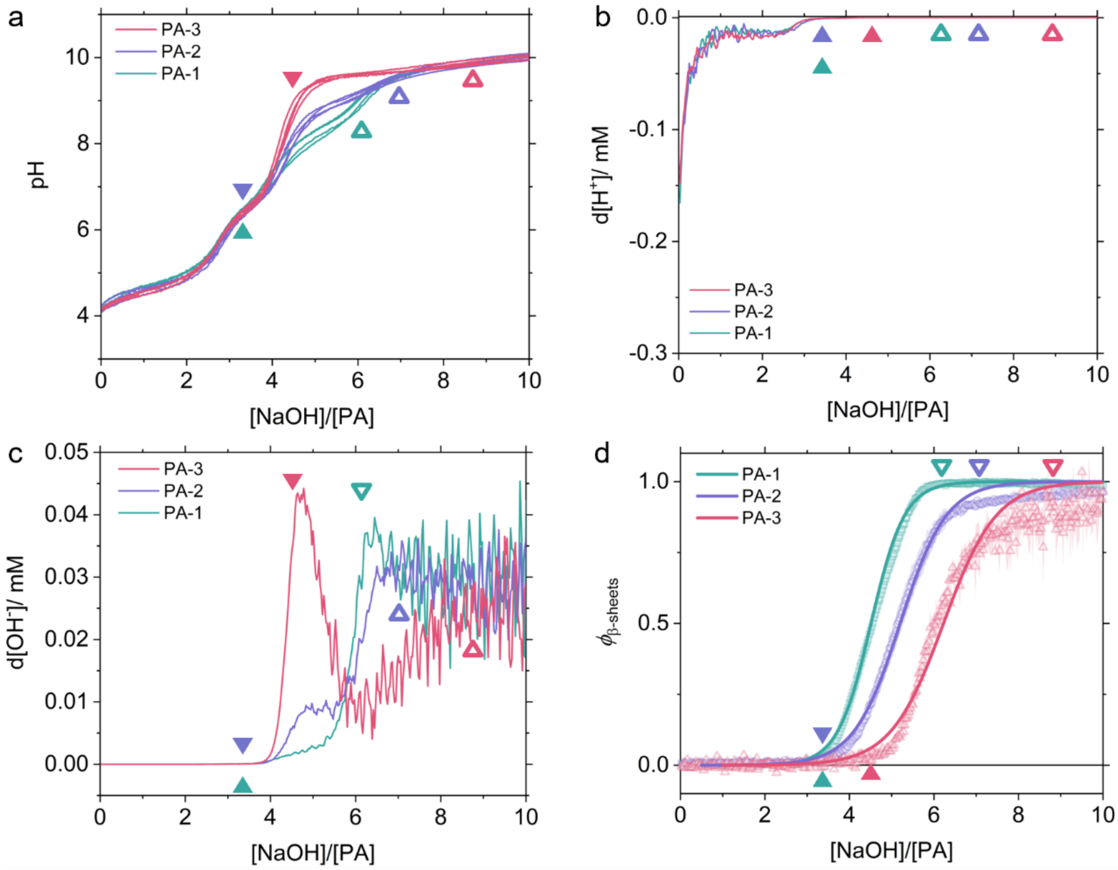

Supplementary Fig. 26. *In-situ* pH titration curves of three PA homopolymerization systems at [PA] = 40 μM. a, pH titration curves monitored *in situ* during the polymerization of PA-1, PA-2, and PA-3 with 4 repetitions for each system. **b-c,** The first derivatives of the **(b)** $[H^+]$ and **(c)** $[OH^-]$ curves. **d,** Reference CD titration curves of three PA systems obtained under the same conditions. Experimentally obtained results are shown in symbols and fits from the *m*-factor mass balance model are shown in lines. Based on these reference CD titration curves, the onsets and ends of polymerization are marked with solid and hollow triangles, respectively, for all the above figures. Measurements were taken from distinct samples (N = 4). Error bars denote the standard deviation (SD).

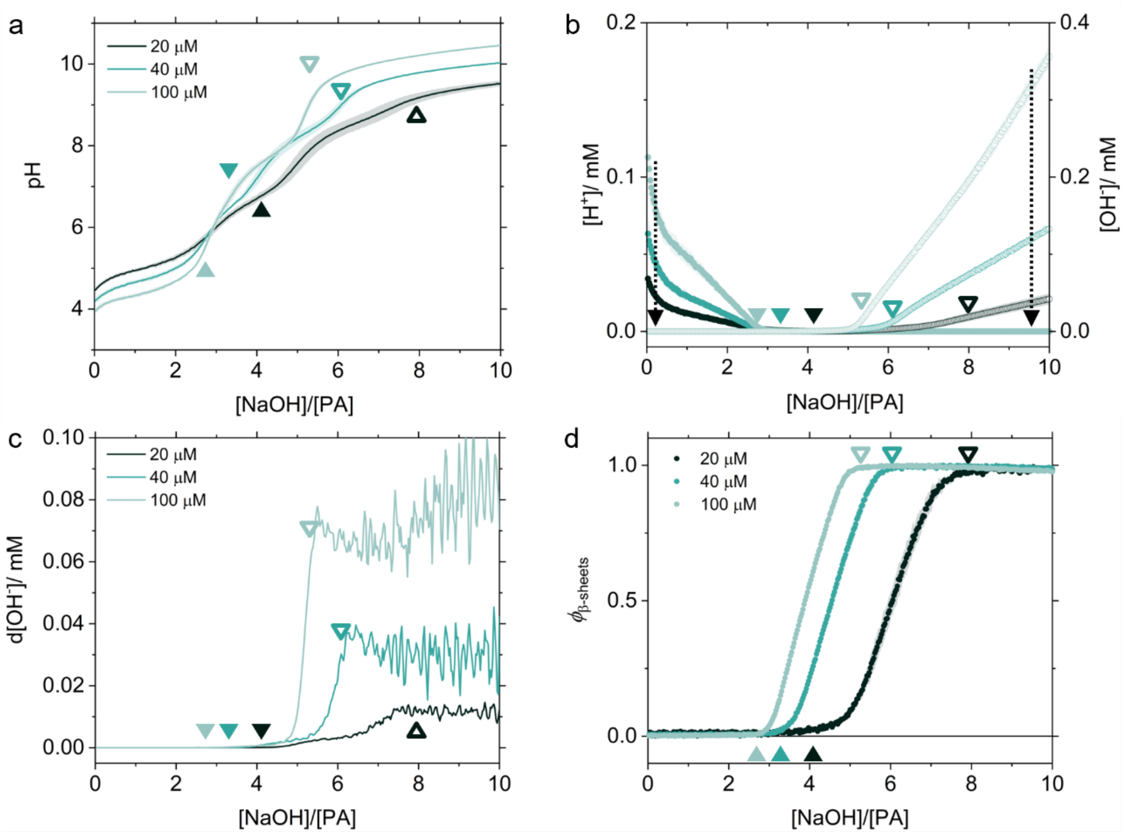

Supplementary Fig. 27. *In-situ* pH titration curves of PA-1 at different [PA]. a, pH titration curves monitored *in situ* during the polymerization of PA-1 at [PA]= 20, 40 and 100 µM. **b,** Concentrations of free protons ([H^+]) (solid symbols) and hydroxide ions ([OH^-]) (empty symbols) in the solution extracted from the pH titration curves. In the direction of arrows, [PA] decreases from 100 to 40 and 20 µM. **c,** The first derivatives of the [OH^-] curves. **d,** Reference CD titration curves obtained under the same conditions. Experimentally obtained results are shown in symbols and fits from the *m*-factor mass balance model are shown in lines. Based on these reference CD titration curves, the onsets and ends of polymerization are marked with solid and hollow triangles, respectively, for the all the above figures. Measurements were taken from distinct samples (N = 4). Error bars denote the standard deviation (SD).

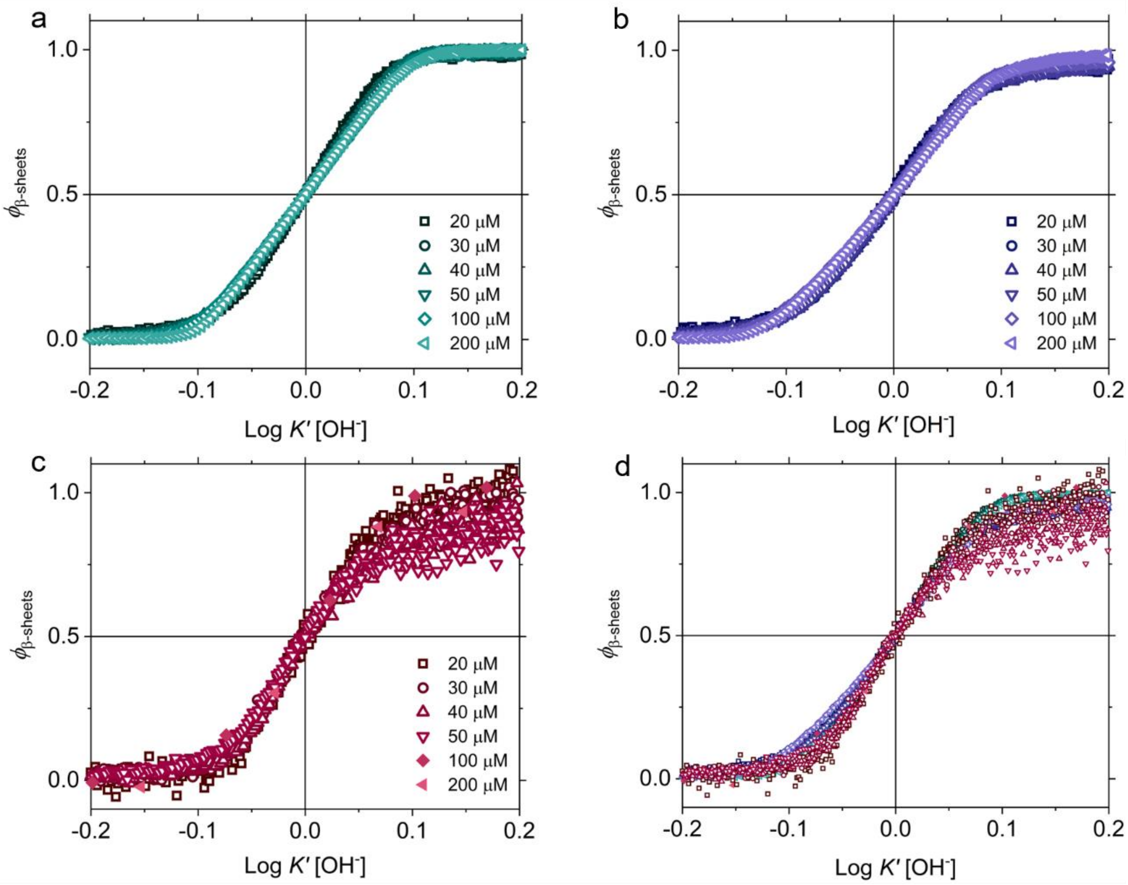

Supplementary Fig. 28. Dependence of $\boldsymbol{\phi_{\beta-\mathbf{sheets}}}$ for three PA homopolymerization systems on the logarithm of the product of the binding constant ($\boldsymbol{K'}$) and the concentration of titrated NaOH ($[\boldsymbol{OH^-}]$) obtained from the allosteric binding model at different [PA]: (a) PA-1, (b) PA-2, (c) PA-3, and (d) the summary of all three PAs. The overlapping curves suggest that all these PA micelles follow the same cooperative deprotonation process to transform into polymers.

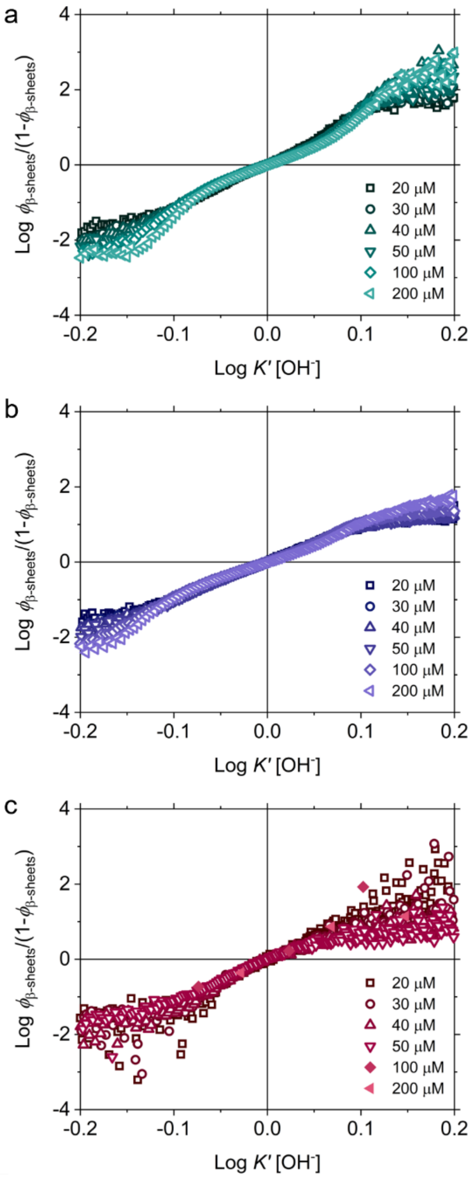

Supplementary Fig. 29. Hill plots for three PA homopolymerization systems extracted from the allosteric binding model: (a) PA-1, (b) PA-2, and (c) PA-3. The Hill plots for each system are consistent at different [PA], indicating that all these micelles follow the same cooperative deprotonation process to transform into polymers.

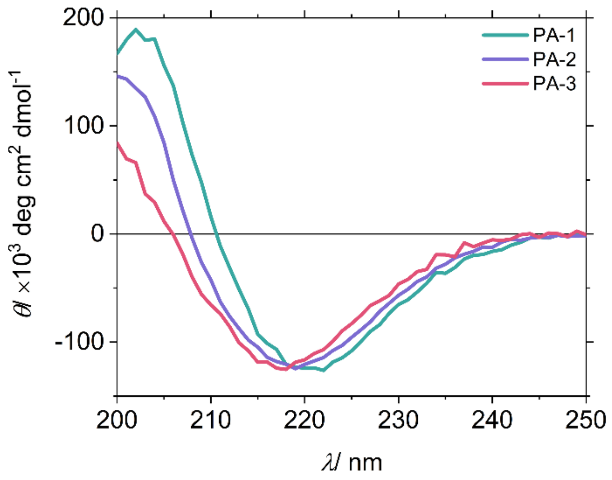

Supplementary Fig. 30. CD spectra of three PA homopolymers formed at [NaOH]/[PA]=15 and [PA] =200 μM. The peak intensity and wavelength are highest in PA-1, decrease in PA-2, and are lowest in PA-3.

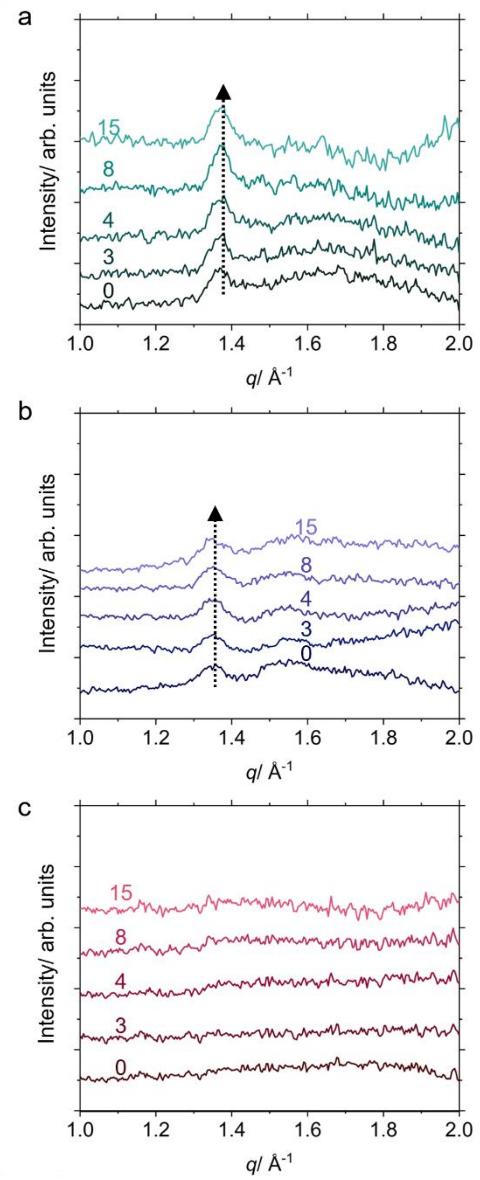

Supplementary Fig. 31. WAXS patterns of three PA homopolymerization systems at different [NaOH]/[PA]: (a) PA-1, (b) PA-2, and (c) PA-3. The concentration of monomers was increased to 2 mM for all systems to ensure a sufficient signal-to-noise ratio in the resulting WAXS patterns. The crystalline diffraction peaks around 1.4 $Å^{-1}$ corresponding to the characteristic *d*-spacing of 4.5 Å for β-sheets are marked by arrows in PA-1 and PA-2 systems.

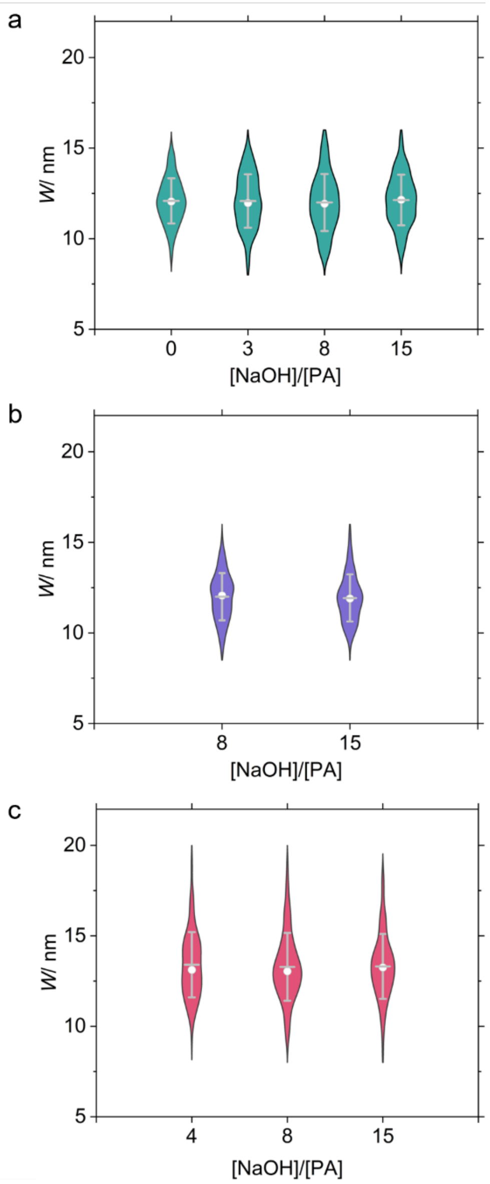

Supplementary Fig. 32. Morphometric analysis of PA homopolymers based on the negative-staining TEM images. a-c, Histograms, mean values with the standard deviation (lines), and median values (circles) of the width of **(a)** PA-1, **(b)** PA-2, and **(c)** PA-3 polymers formed at various [NaOH]/[PA] ratios. These results are further detailed in Supplementary Table 6 to 8. Within each homopolymerization system, the width of formed polymers remains constant at different [NaOH]/[PA] ratios. Error bars denote the standard deviation (SD).

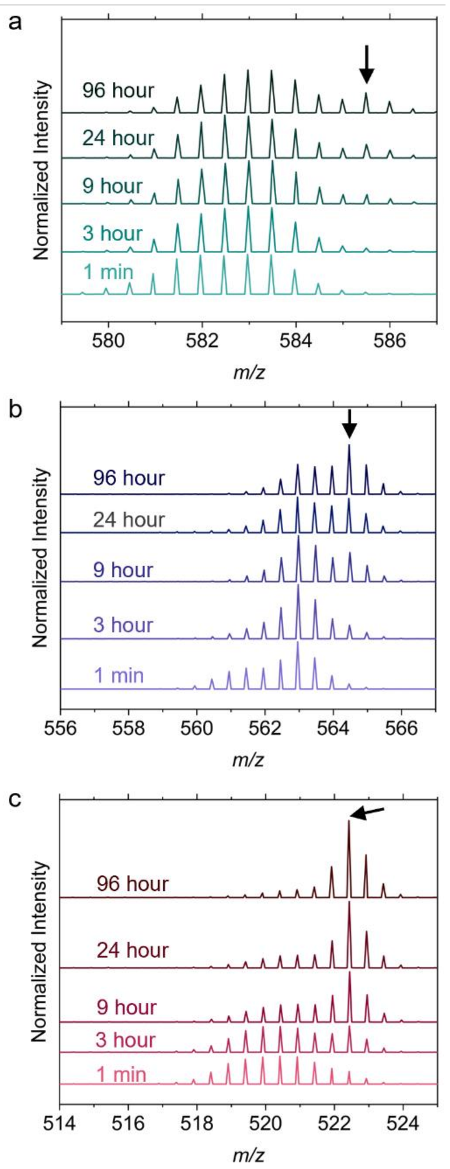

Supplementary Fig. 33. Representative raw HDX-MS spectra of three PA homopolymers at different exchange time: (a) PA-1, (b) PA-2, and (c) PA-3. The spectra are normalized by dividing each individual peak intensity by the sum of the intensity of all displayed peaks. The peaks corresponding to the *m/z* value of fully exchanged species ($[PA17D + 2D]^{2+}$ or abbreviated as PA17D are marked with arrows.

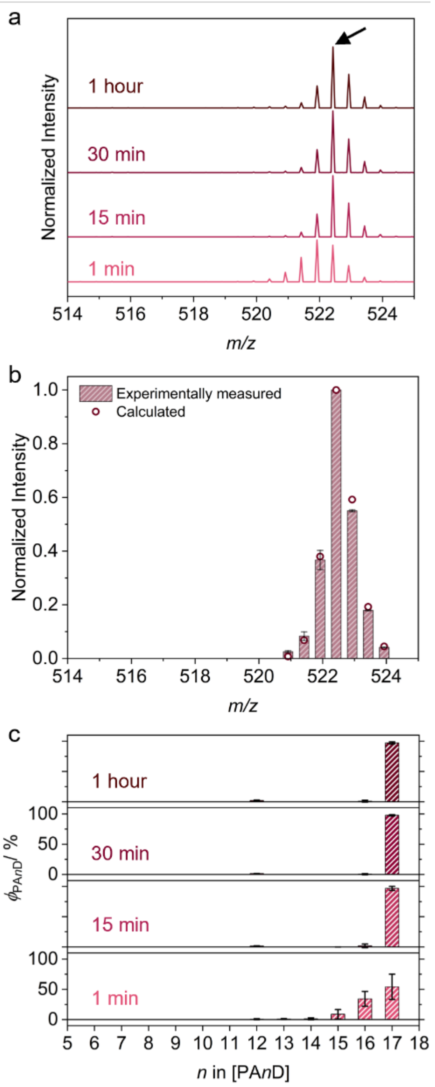

Supplementary Fig. 34. HDX-MS exchange dynamics of PA-3 micelles. a, Representative HDX-MS spectra of PA-3 molecules after exchange in D_2O for different time. The spectra are normalized by dividing each individual peak intensity by the sum of the intensity of all the peaks with *m/z* ranging from 514 to 525. The spectra obtained after 15 min remain similar, in which the highest peak corresponds to the fully exchanged PA17D species at *m/z* = 522.42 (arrow). Peaks at lower *m/z* are attributed to the presence of water in the solution, whereas those at higher *m/z* are contributed by the natural abundance of constituent elements. **b,** Comparison of experimentally obtained MS peaks after an exchange time of 1 h (bars) and fits (circles) considering the presence of water (2 mol%) and natural abundance of elements, as detailed in Supplementary Table 12. The data are normalized by dividing their intensity by that of the peak at *m/z* = 522.42. **c,** Distribution of the molar fractions of various exchanged species ($\phi_{\mathrm{PA}n\mathrm{D}}$) containing *n* exchanged deuterium atoms at different exchange time. The contributions from water and natural abundance of elements of other exchanged species were deconvoluted from the original MS signal of PA*n*D using the Matlab® function lsqnonneg with the prefactors listed in Supplementary Table 12. Measurements were taken from distinct samples (*N* = 3). Error bars denote the standard deviation (SD).

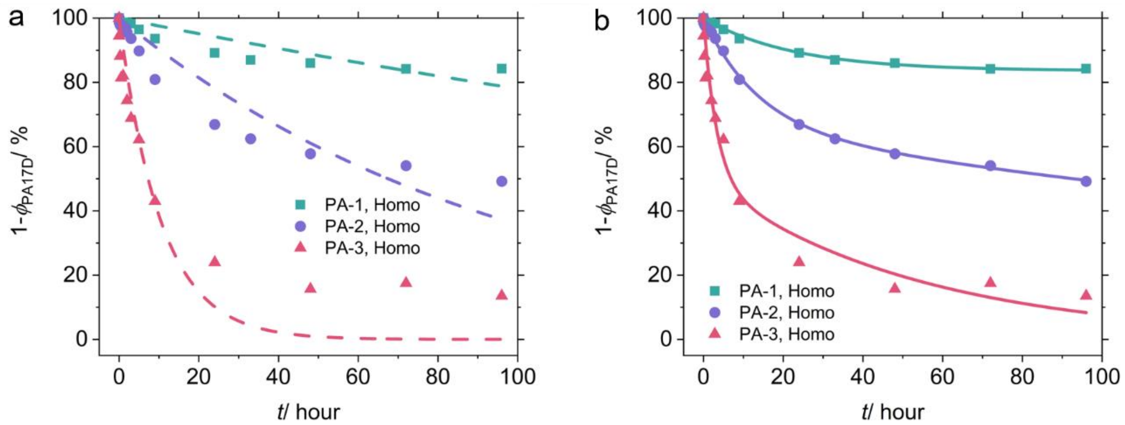

Supplementary Fig. 35. Fits of HDX-MS exchange kinetic curves of three PA homopolymers. a-b, Experimental data (symbols) are fitted with **(a)** a mono-exponential function (dashed lines) and **(b)** a bi-exponential function (solid lines), as detailed in Supplementary Table 13 and 14. The fitting quality with the bi-exponential function is significantly better than the mono-exponential counterpart, as supported by their different R^2 values.

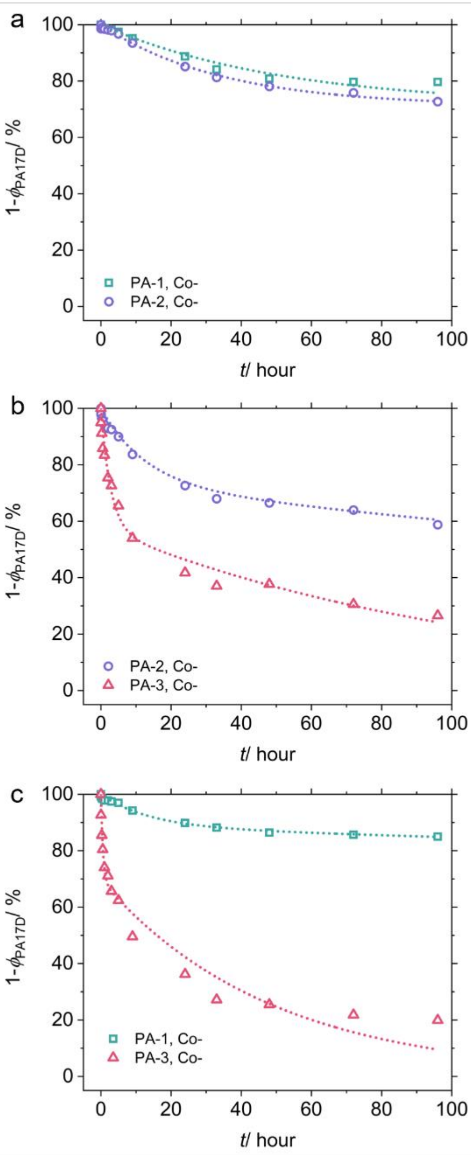

Supplementary Fig. 36. Fits of HDX-MS exchange kinetic curves of three PA copolymers formed at *m.e.* = 0%. a-c, Experimental data (symbols) are fitted with a bi-exponential function (dashed lines) for **(a)** PA-1/2, **(b)** PA-2/3, and **(c)** PA-1/3 copolymers, as detailed in Supplementary Table 15 to 17.

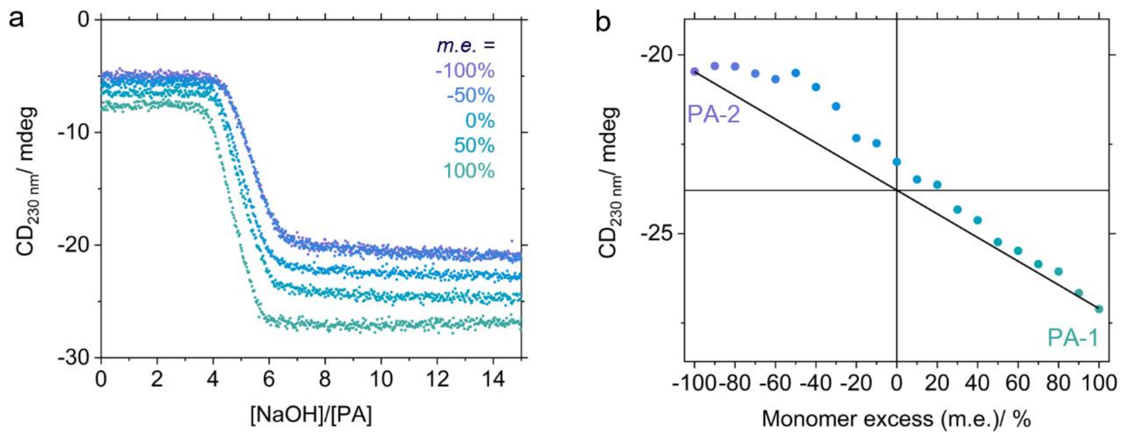

Supplementary Fig. 37. Probing of PA-1/2 copolymerization system using CD titration curves measured at *λ* = 230 nm. a, Representative titration curves measured with different monomer excess (*m.e.*), while $[PA]_{total}$ is maintained at 40 µM. **b,** CD intensity of copolymers extracted at [NaOH]/[PA] = 12 as a function of *m e.*. The linear combination of the CD intensity of respective homopolymers is indicated by the black line. The nonlinear behavior in the CD signal of resulting PA-1/2 copolymers is pronounced, agreeing well with the results obtained at *λ* = 220 nm (Fig. 5a).

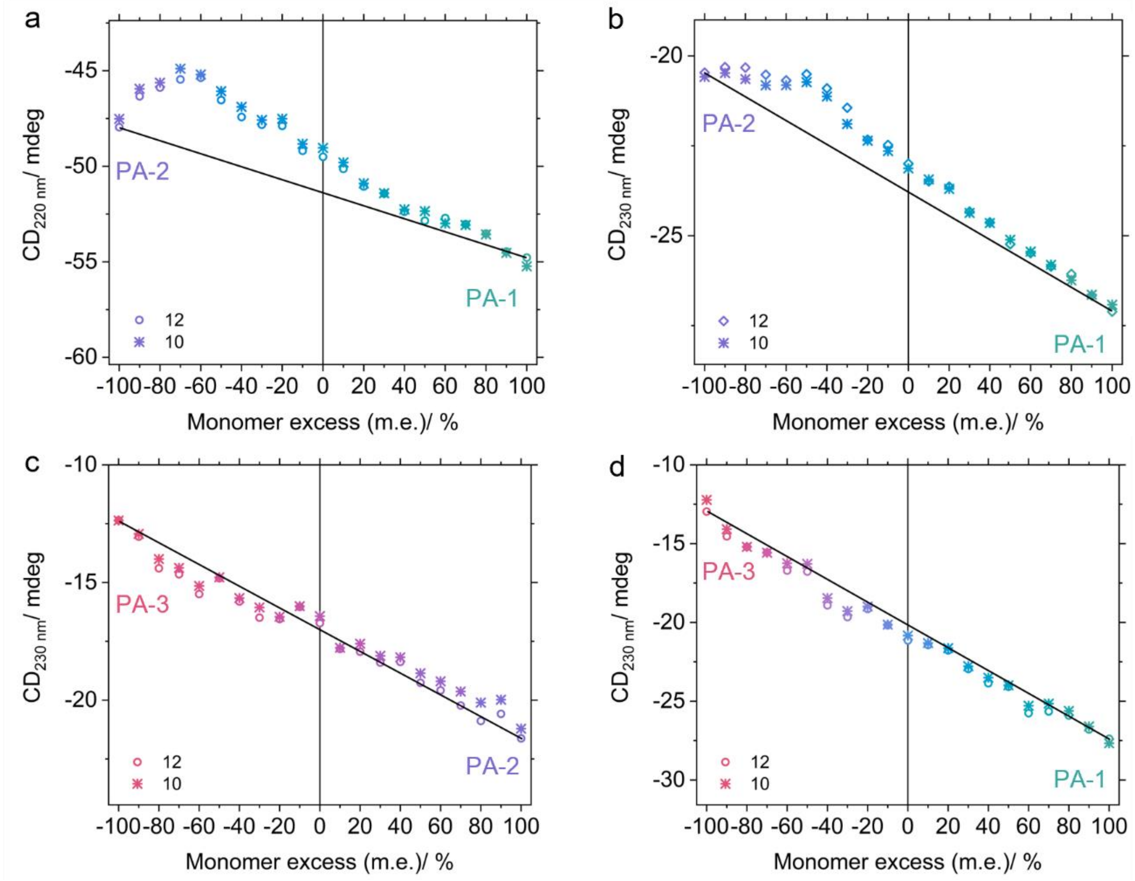

Supplementary Fig. 38. Influence of [NaOH]/[PA] ratio, 10 or 12, at which CD intensity is extracted from the titration curves. a-d, CD intensity as a function of *m. e.* for **(a)** PA-1/2 monitored at 220 nm, **(b)** PA-1/2 monitored at 230 nm, **(c)** PA-2/3 monitored at 230 nm, and **(d)** PA-1/3 monitored at 230nm. Both sets of results are consistent with each other.

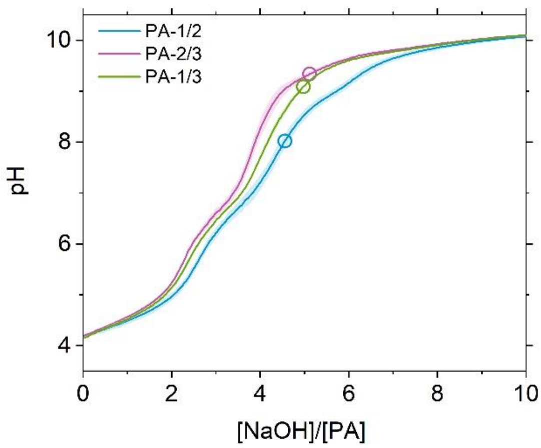

Supplementary Fig. 39. ***In-situ* pH titration curves of copolymerization systems.** *m.e.* = 0%, $[PA]_{total}$ = 40 µM. The apparent pKa values for the PA-1/2, PA-2/3, and PA-1/3 systems are 8.06, 9.34, and 9.09, respectively, which are marked with hollow circles. Measurements were taken from distinct samples (*N* = 3). Error bars denote the standard deviation (SD).

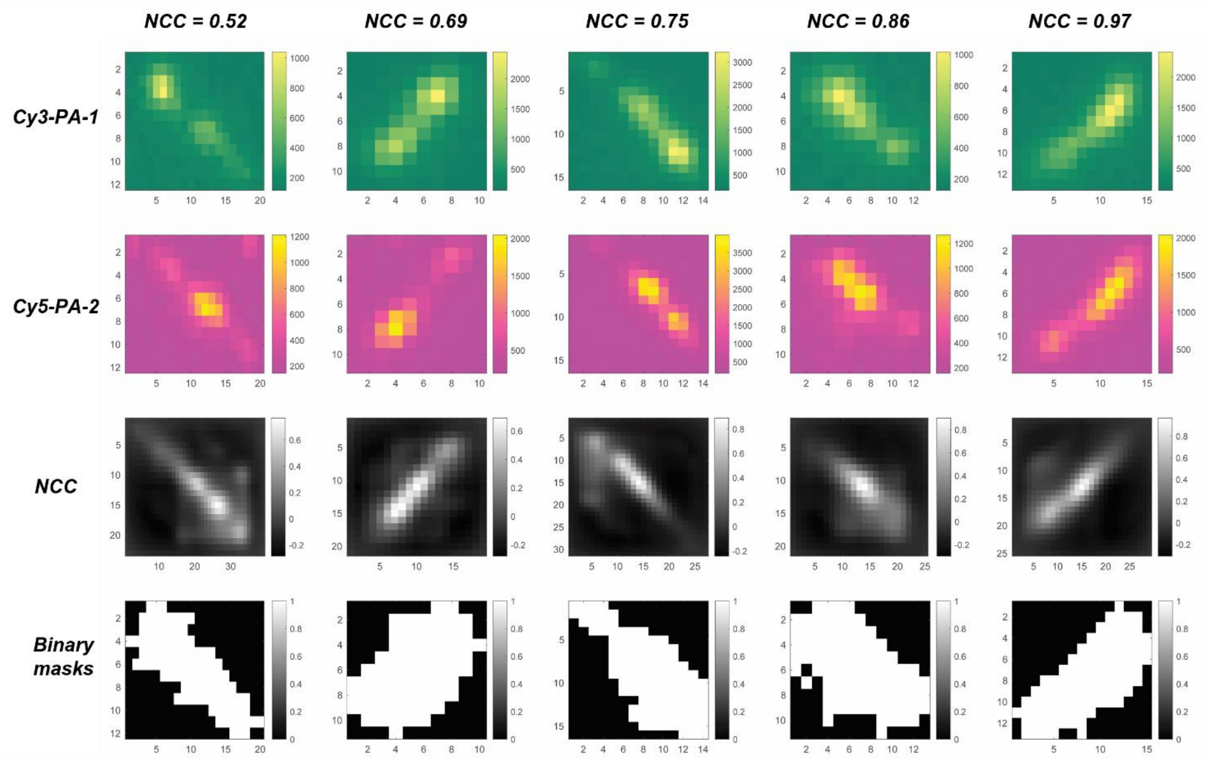

Supplementary Fig. 40. Representative pixeled TIRF microscopy images of PA-1/2 copolymers with different normalized cross-correlation coefficients (NCC) between Cy-3 and Cy-5 channels. 5 mol% of PA-1 monomers are labelled with Cy3 dyes and 5 mol% of PA-2 monomers with Cy5 dyes. The co-polymers are formed at $[PA]_{total}$ = 40 µM and *m.e.* = 0%.

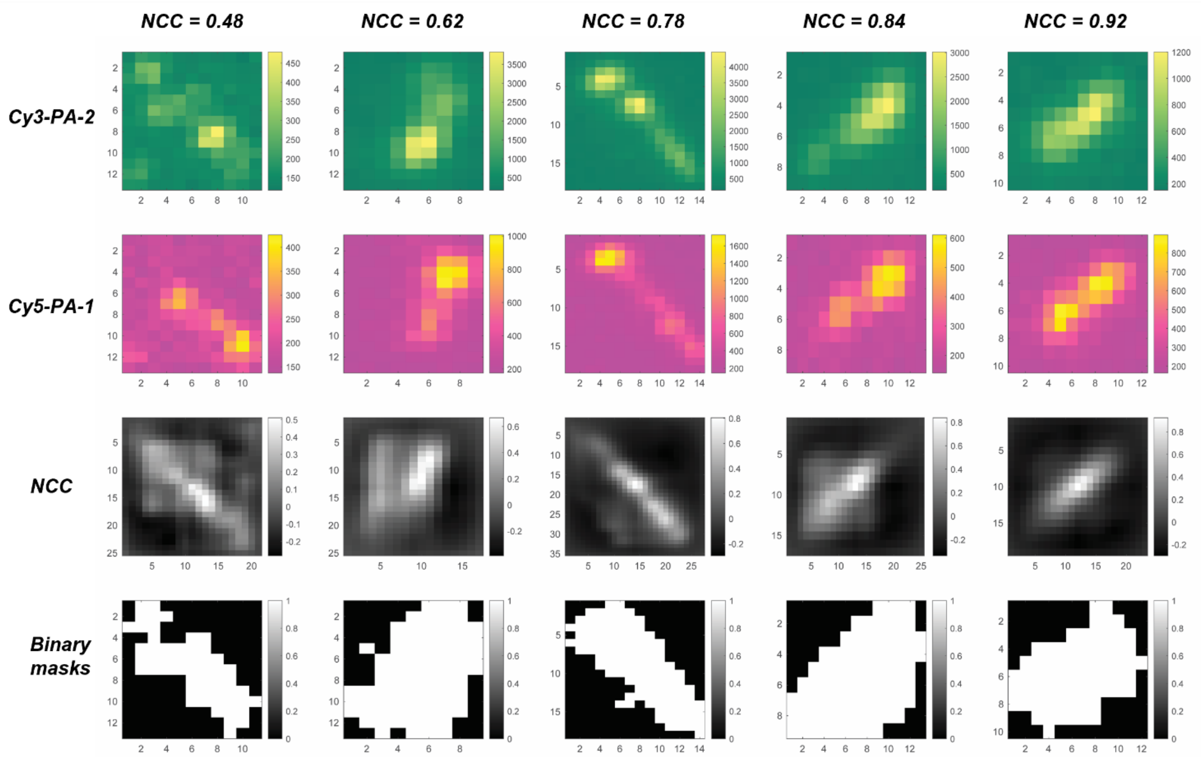

Supplementary Fig. 41. Representative pixeled TIRF microscopy images of PA-1/2 copolymers with different NCC values between Cy-3 and Cy-5 channels. 5 mol% of PA-2 monomers are labelled with Cy3 dyes and 5 mol% of PA-1 monomers with Cy5 dyes. The co-polymers are formed at $[PA]_{total}$ = 40 μM and *m.e.* = 0%.

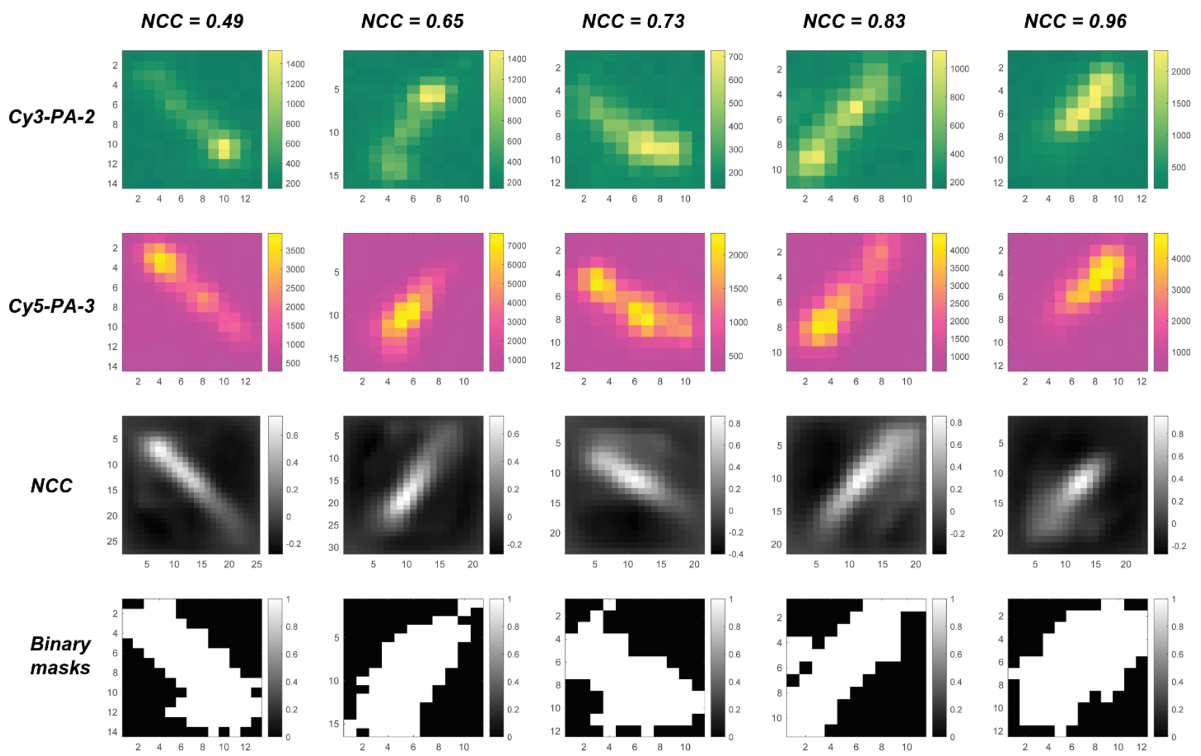

Supplementary Fig. 42. Representative pixeled TIRF microscopy images of PA-2/3 copolymers with different NCC values between Cy-3 and Cy-5 channels. 5 mol% of PA-2 monomers are labelled with Cy3 dyes and 5 mol% of PA-3 monomers with Cy5 dyes. The co-polymers are formed at $[PA]_{total}$ = 40 µM and *m.e.* = 0%.

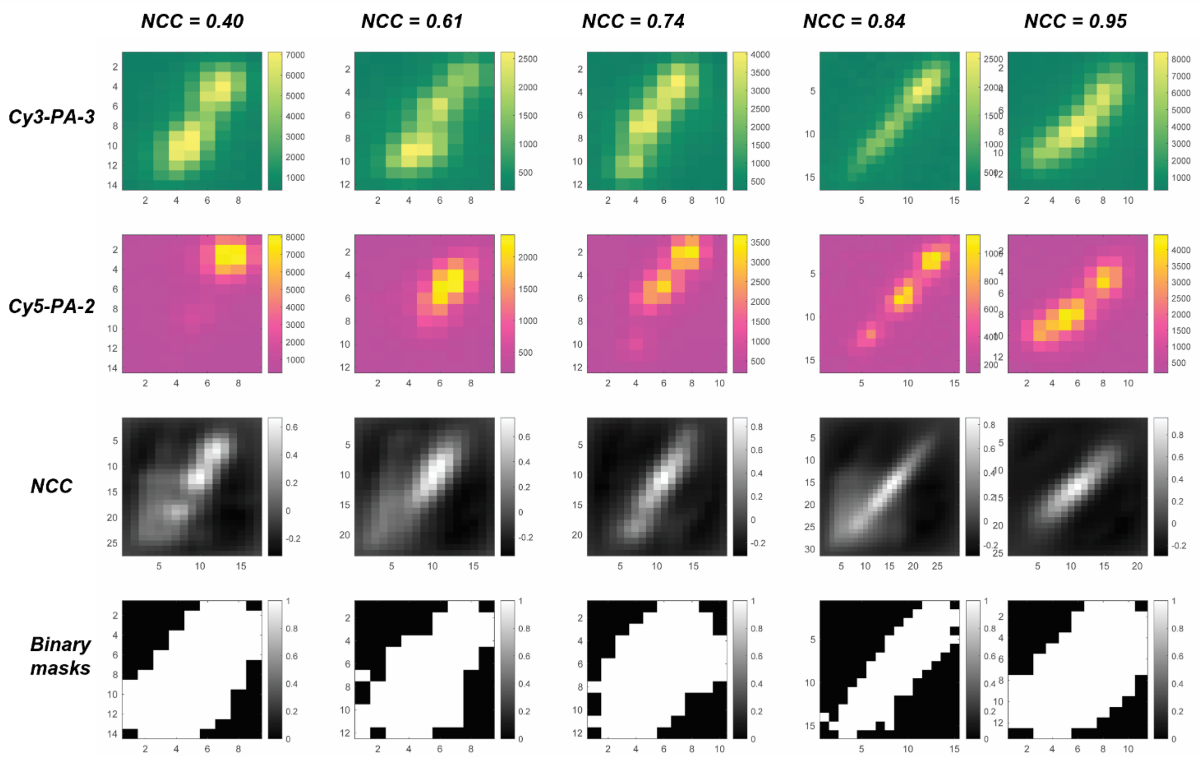

Supplementary Fig. 43. Representative pixeled TIRF microscopy images of PA-2/3 copolymers with different NCC values between Cy-3 and Cy-5 channels. 5 mol% of PA-3 monomers are labelled with Cy3 dyes and 5 mol% of PA-2 monomers with Cy5 dyes. The co-polymers are formed at $[PA]_{total}$ = 40 µM and *m.e.* = 0%.

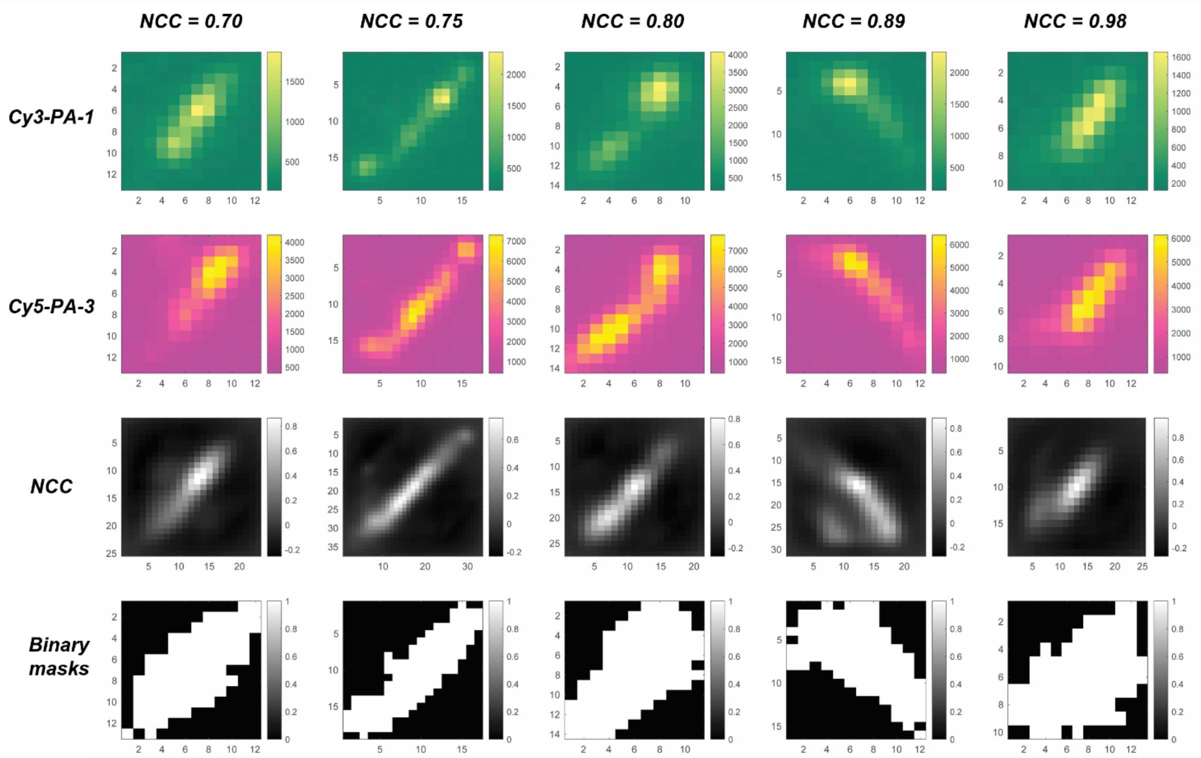

Supplementary Fig. 44. Representative pixeled TIRF microscopy images of PA-1/3 copolymers with different NCC values between Cy-3 and Cy-5 channels. 5 mol% of PA-1 monomers are labelled with Cy3 dyes and 5 mol% of PA-3 monomers with Cy5 dyes. The co-polymers are formed at $[PA]_{total}$ = 40 µM and *m.e.* = 0%.

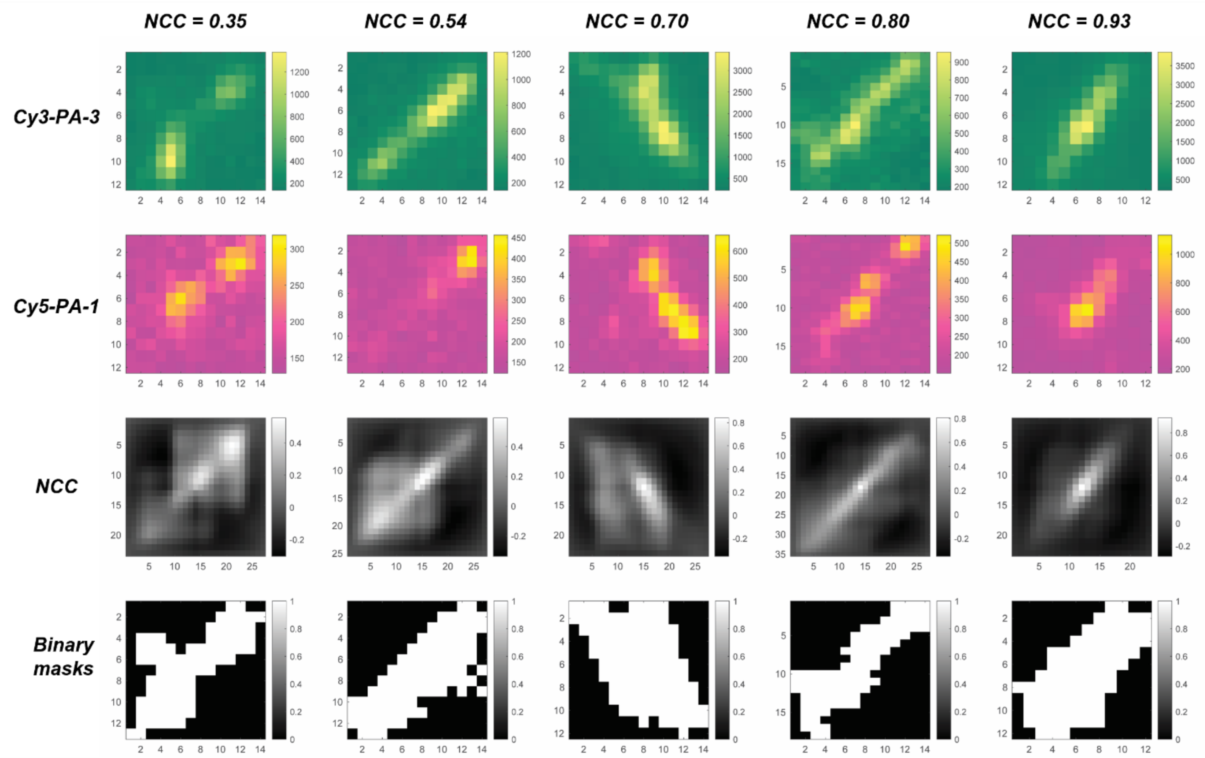

Supplementary Fig. 45. Representative pixeled TIRF microscopy images of PA-1/3 copolymers with different NCC values between Cy-3 and Cy-5 channels. 5 mol% of PA-3 monomers are labelled with Cy3 dyes and 5 mol% of PA-1 monomers with Cy5 dyes. The co-polymers are formed at $[PA]_{total}$ = 40 µM and *m.e.* = 0%.

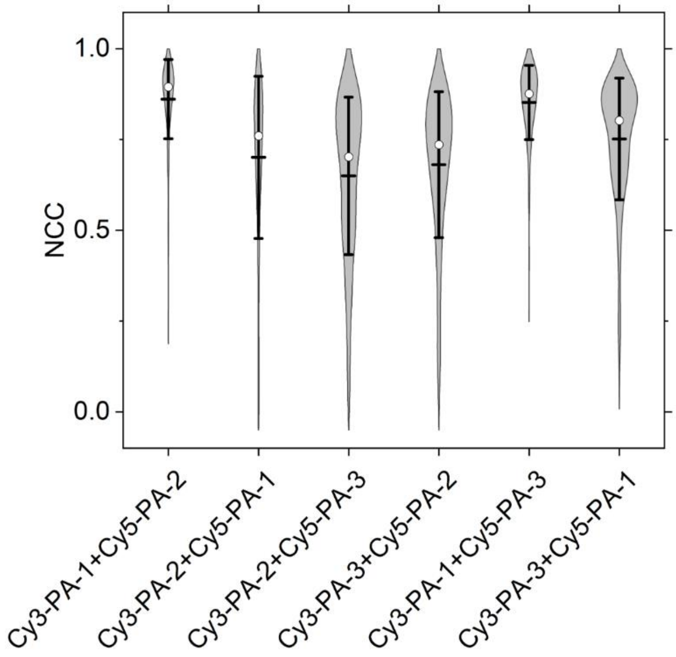

Supplementary Fig. 46. Cross-correlation analysis of TIRF images of PA copolymers formed at *m.e.* = 0%. Histograms, mean values with the standard deviation (lines), and median values (circles) of the normalized cross-correlation coefficients between Cy3 and Cy5 channels in the TIRF images of different PA copolymers, in which 5 mol% of constituent monomers are covalently labeled with Cy3 or Cy5 fluorescent dyes. *N* values for the systems shown along the X-axis from the left to right are 145, 112, 337, 345, 219, and 473, respectively. Error bars denote the standard deviation (SD).

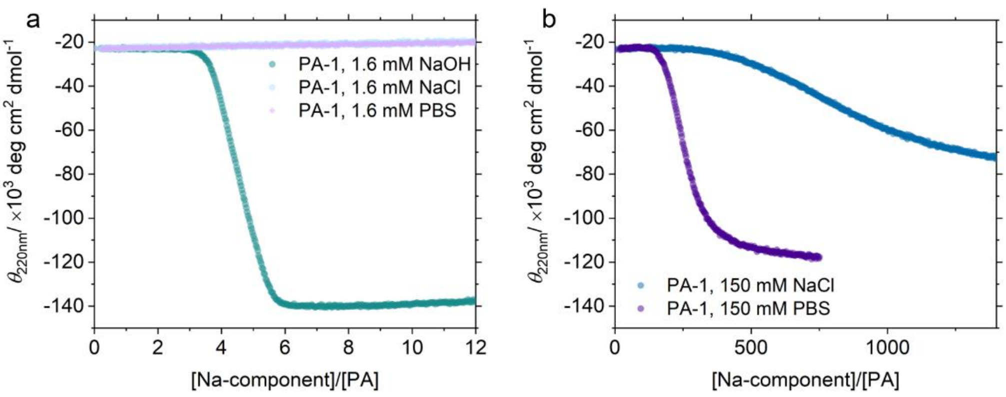

Supplementary Fig. 47. Demonstration of *In-situ* CD titration curves of PA-1 with NaCl and PBS as titrants, [PA] = 40 μM. (a) Titration curves up to a molar ratio of [Na-component]/[PA] = 12 using 1.6 mM NaCl and 1.6 mM PBS. The titration curve with 1.6 mM NaOH is included for comparison. (b) Titration curves up to [Na-component]/[PA] = 1400 using 150 mM NaCl and 150 mM PBS. For the X-axis, the commercially available 1× PBS is approximated to contain 150 mM Na-based components during the preparation of the PBS titrant solutions. Notably, the efficiency in driving the polymerization of PA-1 decreases in the order of NaOH, PBS, and NaCl. This result indicates the deprotonation process is more effective than the charge screening due to elevated ionic strengths of the solution to drive the supramolecular polymerization of PA-1.

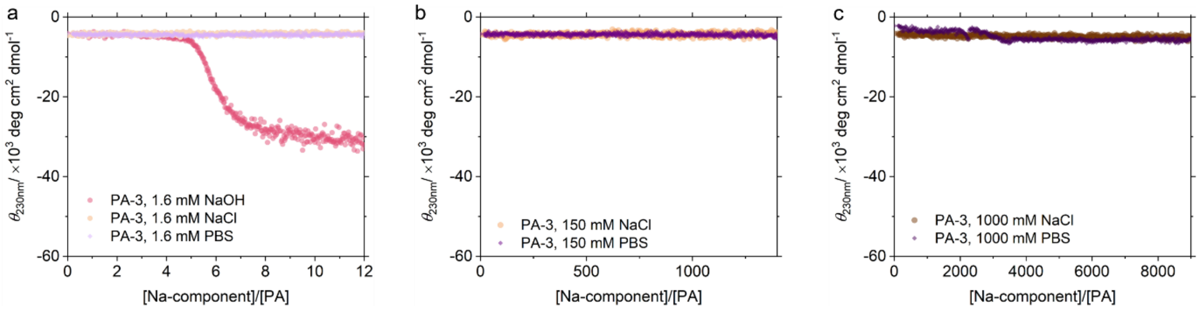

Supplementary Fig. 48. Demonstration of *In-situ* CD titration curves of PA-3 with NaCl and PBS as titrants, [PA] = 40 μM. (a) Titration curves up to a molar ratio of (a) [Na-component]/[PA] = 12 using 1.6 mM NaCl and 1.6 mM PBS. The titration curve with 1.6 mM NaOH is included for comparison. (b) Titration curves up to [Na-component]/[PA] = 1400 using 150 mM NaCl and 150 mM PBS. (c) Titration curves up to [Na-component]/[PA] = 9300 using 1000 mM NaCl and 1000 mM PBS. For the X-axis, the commercially available 10× PBS and 1× PBS are approximated to contain 1500 mM and 150 mM Na-based components, respectively. Consistently, NaOH displays highest efficiency in driving the polymerization of PA-3, significantly higher than PBS and NaCl. Surprisingly, PBS or NaCl at a molar ratio of [Na-component]/[PA] as high as 9300 does not initiate the polymerization of PA-3, underscoring the dominant role of deprotonation over charge screening in certain PA systems.

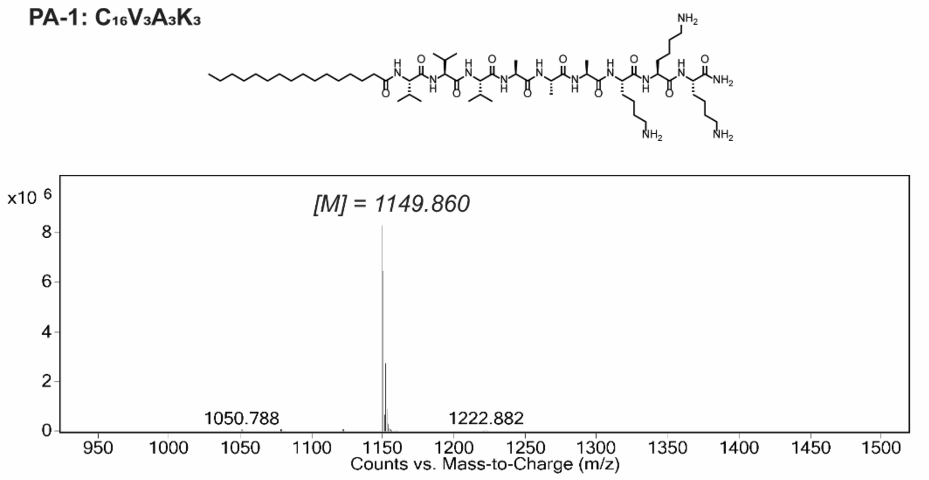

Supplementary Fig. 49. Chemical structure (top) and mass spectrum (bottom) of PA-1: $C_{16}V_3A_3K_3$. The purity is higher than 95%.

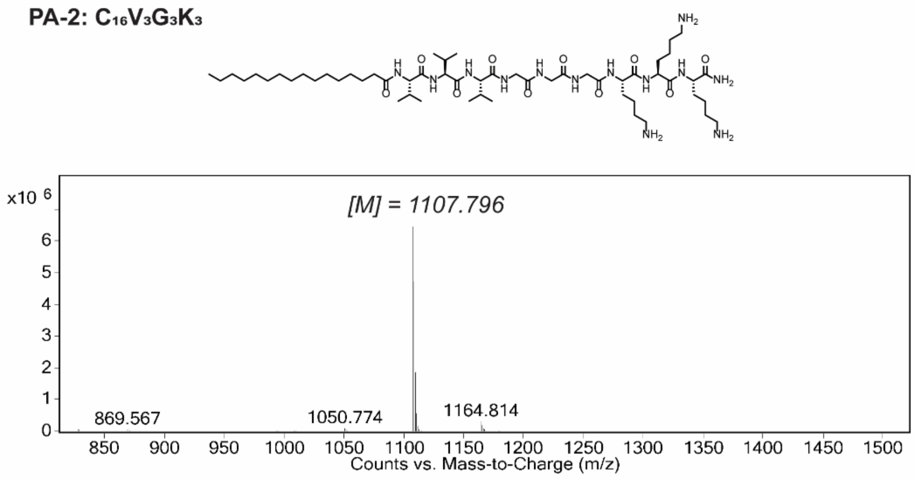

Supplementary Fig. 50. Chemical structure (top) and mass spectrum (bottom) of PA-2: $C_{16}V_3G_3K_3$. The purity is higher than 95%.

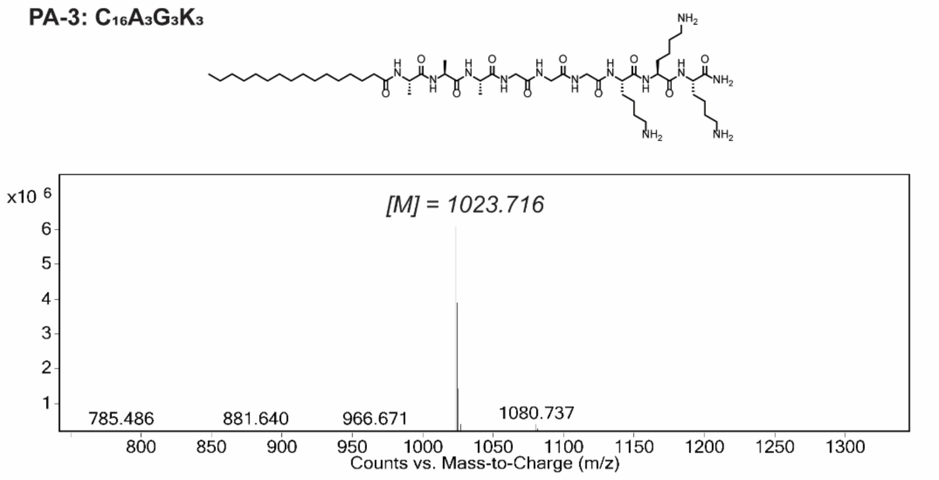

Supplementary Fig. 51. Chemical structure (top) and mass spectrum (bottom) of PA-3: $C_{16}A_3G_3K_3$. The purity is higher than 95%.

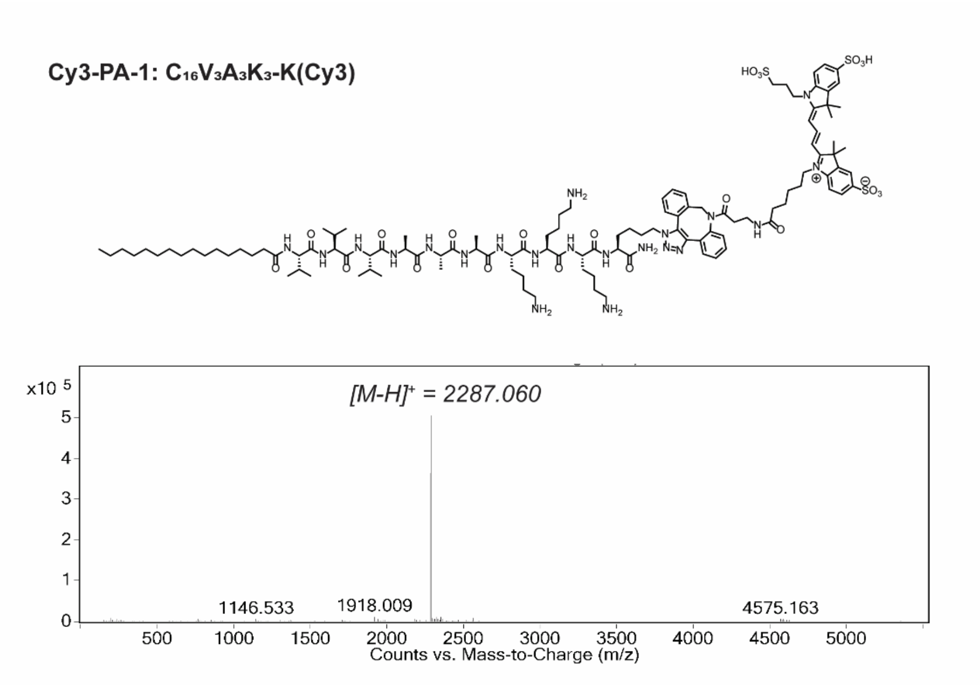

Supplementary Fig. 52. Chemical structure (top) and mass spectrum (bottom) of Cy3-PA-1: $C_{16}V_3A_3K_3$-K(Cy3). The purity is higher than 95%.

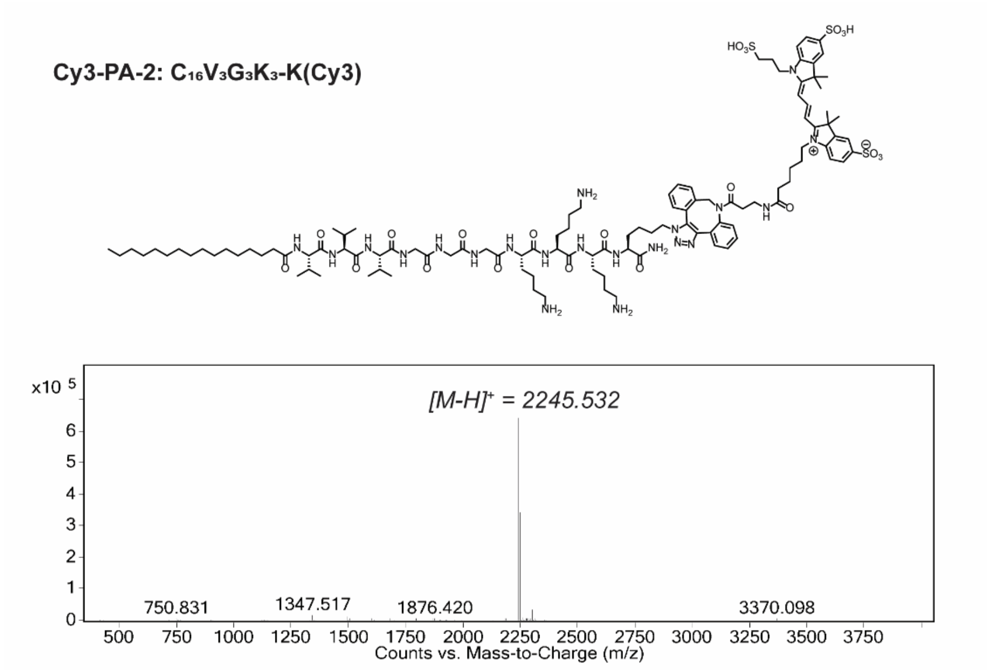

Supplementary Fig. 53. **Chemical structure (top) and mass spectrum (bottom) of Cy3-PA-2: $C_{16}V_3G_3K_3$-K(Cy3).** The purity is higher than 95%.

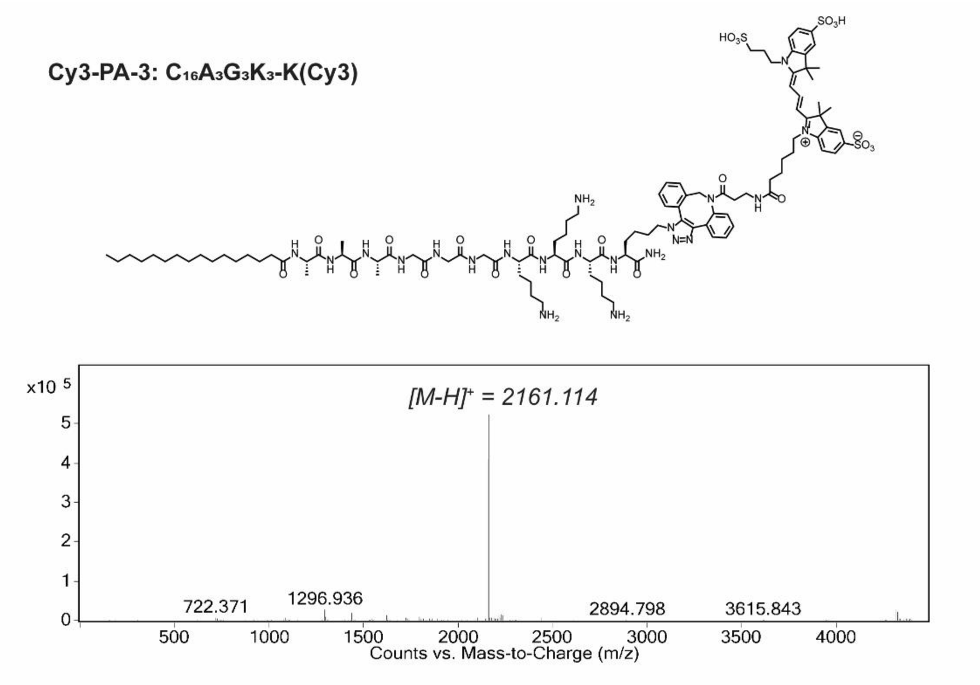

Supplementary Fig. 54. Chemical structure (top) and mass spectrum (bottom) of Cy3-PA-3: $C_{16}A_3G_3K_3$-K(Cy3). The purity is higher than 95%.

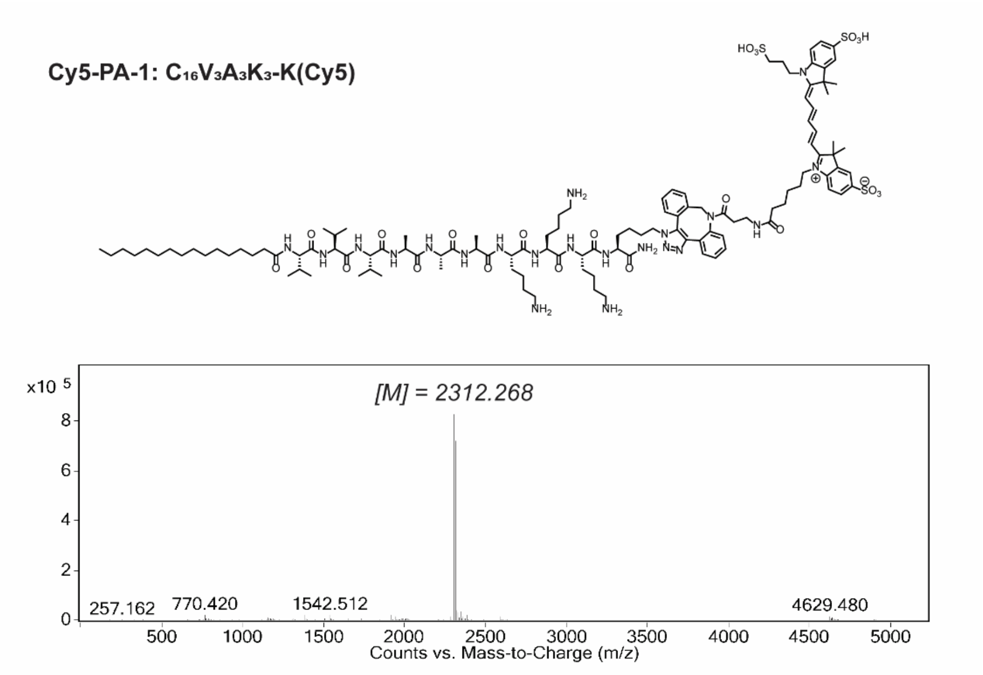

Supplementary Fig. 55. Chemical structure (top) and mass spectrum (bottom) of Cy5-PA-1: $C_{16}V_3A_3K_3$-K(Cy5). The purity is higher than 95%.

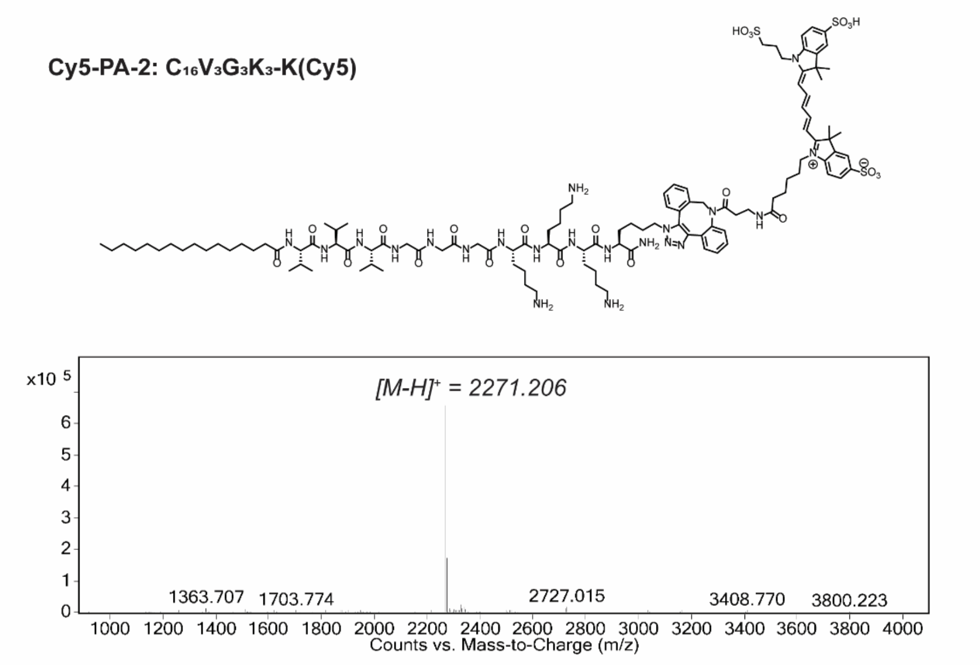

Supplementary Fig. 56. Chemical structure (top) and mass spectrum (bottom) of Cy5-PA-2: $C_{16}V_3G_3K_3$-K(Cy5). The purity is higher than 95%.

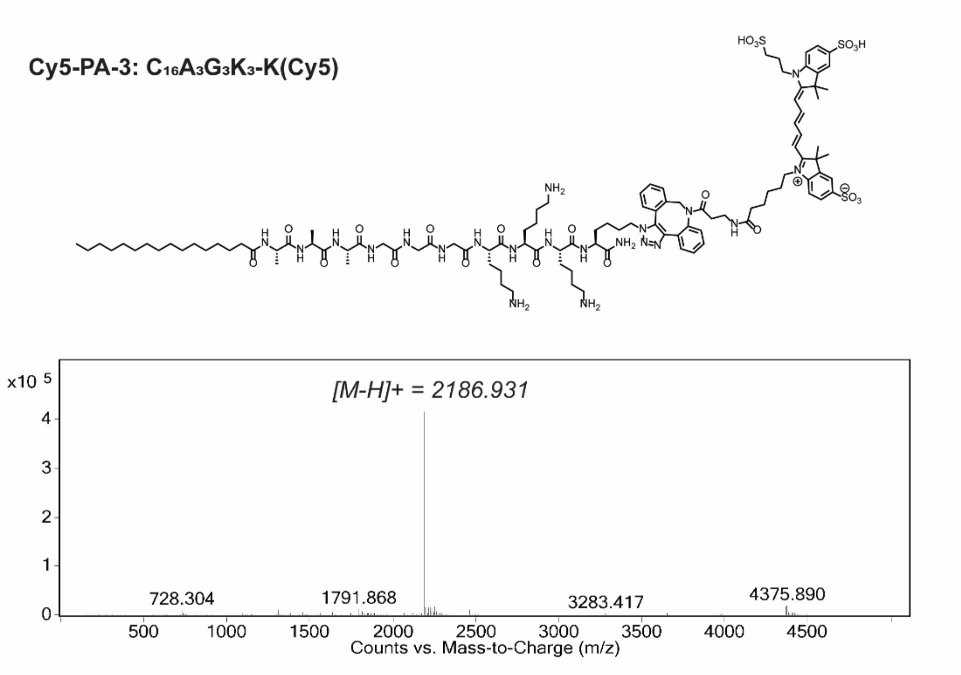

Supplementary Fig. 57. Chemical structure (top) and mass spectrum (bottom) of Cy5-PA-3: $C_{16}A_3G_3K_3$-K(Cy5). The purity is higher than 95%.

Tables

[PA]/ µM	[NaOH]/ mM
4000	160
2000	80
1000	40
500	20
200	8
100	4
50	2
40	1.6
30	1.2
20	0.8

Supplementary Table 1. Concentrations of PA and NaOH solutions used in our work. To ensure the same dilution effect during titration, the same concentration ratio between the two solutions was kept.

[PA]/ µM	A_1	A_2	x_0	R^2
1000	-7.28 × 10^4	-1.52 × 10^5	3.38	0.99
500	-4.16 × 10^4	-1.26 × 10^5	3.29	0.98

Supplementary Table 2. Parameters for the Boltzmann fits of the titration curves of PA-1 with the equation:

$$\theta = \frac{A_1 - A_2}{1 + e^{(x-x_0)/dx}} + A_2$$

[PA]/ µM	A_1	A_2	x_0	R^2
1000	-3.96 × 10^4	-1.16 × 10^5	3.28	0.98
500	-1.69 × 10^4	-1.19 × 10^5	3.53	0.96

Supplementary Table 3. Parameters for the Boltzmann fits of the titration curves of PA-2.

[PA]/ µM	A_1	A_2	x_0	R^2
2000	-2.70×10^3	-4.70×10^4	3.63	0.99
1000	-2.57×10^3	-4.72×10^4	3.74	0.99
500	-2.86×10^3	-4.67×10^4	3.86	0.99
200	-3.19×10^3	-4.45×10^4	4.25	0.99
100	-3.25×10^3	-4.27×10^4	4.78	0.99

Supplementary Table 4. Parameters for the Boltzmann fits of the titration curves of PA-3 measured at the wavelength of 230 nm.

[PA]/ µM	A_1	A_2	x_0	R^2
2000	1.89×10^3	-1.00×10^5	3.35	0.99
1000	1.80×10^3	-1.07×10^5	3.61	0.99
500	1.54×10^3	-1.11×10^5	3.81	0.99
200	1.26×10^3	-1.12×10^5	4.04	0.99
100	1.13×10^3	-1.09×10^5	4.73	0.99

Supplementary Table 5. Parameters for the Boltzmann fits of the titration curves of PA-3 measured at the wavelength of 220 nm.

[NaOH]/[PA]	PA-1 width/ nm	Counts
0	12.1 ± 1.3 nm	166
3	12.1 ± 1.5 nm	155
8	12.0 ± 1.6 nm	168
15	12.1 ± 1.4 nm	174

Supplementary Table 6. Mean values and standard deviation of the width of PA-1 polymers formed at different [NaOH]/[PA] ratios.

[NaOH]/[PA]	PA-2 width/ nm	Counts
8	12.0 ± 1.3 nm	176
15	11.9 ± 1.3 nm	193

Supplementary Table 7. Mean values and standard deviation of the width of PA-2 polymers formed at different [NaOH]/[PA] ratios.

[NaOH]/[PA]	PA-3 width/ nm	Counts
4	13.4 ± 1.8 nm	125
8	13.3 ± 1.9 nm	181
15	13.3 ± 1.8 nm	148

Supplementary Table 8. Mean values and standard deviation of the width of PA-3 polymers formed at different [NaOH]/[PA] ratios.

[PA]/ μM	m/ kJ mol^{-1}	ΔG_0/ kJ mol^{-1}
20	-4.58	27.70
30	-5.73	
40	-6.14	
50	-6.22	
100	-7.09	
200	-7.63	

Supplementary Table 9. Thermodynamic parameters for the fits of the normalized CD titration curves of PA-1 using *m*-factor mass balance model.

[PA]/ μM	m/ kJ mol^{-1}	ΔG_0/ kJ mol^{-1}
20	-3.23	23.08
30	-4.05	
40	-4.44	
50	-4.74	
100	-5.57	
200	-6.08	

Supplementary Table 10. Thermodynamic parameters for the fits of the normalized CD titration curves of PA-2 using *m*-factor mass balance model.

[PA]/ μM	m/ kJ mol^{-1}	ΔG_0/ kJ mol^{-1}
20	-2.50	24.21
30	-3.34	
40	-3.89	
50	-4.49	
100	-5.11	
200	-5.61	

Supplementary Table 11. Thermodynamic parameters for the fits of the normalized CD titration curves of PA-3 using *m*-factor mass balance model.

	Isotope	PA-1	PA-2	PA-3
Contribution of H_2O	$\phi_{[\mathrm{PA}n\mathrm{D}]-3}$	0.008	0.008	0.008
	$\phi_{[\mathrm{PA}n\mathrm{D}]-2}$	0.068	0.068	0.068
	$\phi_{[\mathrm{PA}n\mathrm{D}]-1}$	0.380	0.380	0.380
	$\phi_{[\mathrm{PA}n\mathrm{D}]}$	1.000	1.000	1.00
Natural abundance	$\phi_{[\mathrm{PA}n\mathrm{D}]+1}$	0.690	0.658	0.592
	$\phi_{[\mathrm{PA}n\mathrm{D}]+2}$	0.256	0.234	0.193
	$\phi_{[\mathrm{PA}n\mathrm{D}]+3}$	0.067	0.059	0.045
	$\phi_{[\mathrm{PA}n\mathrm{D}]+4}$	0.014	0.012	0.008

Supplementary Table 12. Theoretical isotopic distribution of exchanged species of [PA*n*D] by considering the natural abundance of elements using IsoPro Software and the contribution of H_2O content ($\phi_{\mathrm{H_2O}} \approx 2$ mol%) in the solution.

$$\phi_{[\mathrm{PA}n\mathrm{D}]-1} = \phi_{[\mathrm{PA}n\mathrm{D}]} \cdot C(19,1) \cdot \phi_{\mathrm{H_2O}} = 0.38$$
$$\phi_{[\mathrm{PA}n\mathrm{D}]-2} = \phi_{[\mathrm{PA}n\mathrm{D}]} \cdot C(19,2) \cdot \phi_{\mathrm{H_2O}}{}^{2} \approx 0.068$$
$$\phi_{[\mathrm{PA}n\mathrm{D}]-3} = \phi_{[\mathrm{PA}n\mathrm{D}]} \cdot C(19,3) \cdot \phi_{\mathrm{H_2O}}{}^{3} \approx 0.008$$

Polymer	k/ hour^{-1}	R^2
PA-1, Homo-	2.5 × 10^{-3}	0.76
PA-2, Homo-	1.0 × 10^{-2}	0.88
PA-3, Homo-	9.5 × 10^{-2}	0.89

Supplementary Table 13. Parameters for the fits of HDX-MS exchange kinetics curves of three PA homopolymers using the mono-exponential function: $1 - \phi_{[\mathrm{PA17D}]} = e^{-k \cdot t}$.

Polymer	k_{fast}/ hour^{-1}	k_{slow}/ hour^{-}	A_1	A_2	R^2
PA-1, Homo-	4.4 × 10^{-2}	*1.0 × 10^{-5}	16.4%	83.6%	0.99
PA-2, Homo-	7.3 × 10^{-2}	3.0 × 10^{-3}	34.2%	65.8%	0.99
PA-3, Homo-	3.0 × 10^{-1}	1.9 × 10^{-2}	50.4%	49.6%	0.96

Supplementary Table 14. Parameters for the fits of HDX-MS exchange kinetics curves of three PA homopolymers using the bi-exponential function: $1 - \phi_{[\mathrm{PA17D}]} = A_1 \cdot e^{-k_{\mathrm{fast}} \cdot t} + A_2 \cdot e^{-k_{\mathrm{slow}} \cdot t}$, where $A_1 + A_2 = 1$. The quality of these fits is substantially better than those obtained with the mono-exponential function. The asterisk symbol denotes that the limited time span of the HDX-MS measurements results in divergent values for the fit parameter of k_{slow} in PA-1. Hence, we manually set a lower bound of 1.0 × 10^{-5} for the k_{slow} parameter here to avoid irrelevant, infinitely small value.

Polymer	k_{fast}/ hour^{-1}	k_{slow}/ hour^{-}	A_1	A_2	R^2
PA-1, Co-	1.9×10^{-2}	$*1.0 \times 10^{-5}$	28.8%	71.2%	0.96
PA-2, Co-	2.9×10^{-2}	$*1.0 \times 10^{-5}$	29.0%	71.0%	0.99

Supplementary Table 15. Parameters for the fits of HDX-MS exchange kinetics curves of PA-1/2 copolymers using the bi-exponential function: $1 - \phi_{[PA17D]} = A_1 \cdot e^{-k_{fast} \cdot t} + A_2 \cdot e^{-k_{slow} \cdot t}$, where $A_1 + A_2 = 1$. The asterisk symbol denotes that a lower bound of 1.0×10^{-5} was set for the k_{slow} parameter.

Polymer	k_{fast}/ hour^{-1}	k_{slow}/ hour^{-}	A_1	A_2	R^2
PA-2, Co-	7.8×10^{-2}	2.0×10^{-3}	26.8%	73.2%	0.97
PA-3, Co-	3.6×10^{-1}	9.0×10^{-3}	42.4%	57.6%	0.97

Supplementary Table 16. Parameters for the fits of HDX-MS exchange kinetics curves of PA-2/3 copolymers using the bi-exponential function: $1 - \phi_{[PA17D]} = A_1 \cdot e^{-k_{fast} \cdot t} + A_2 \cdot e^{-k_{slow} \cdot t}$, where $A_1 + A_2 = 1$.

Polymer	k_{fast}/ hour^{-1}	k_{slow}/ hour^{-}	A_1	A_2	R^2
PA-1, Co-	7.2×10^{-2}	4.4×10^{-4}	11.5%	88.5%	0.98
PA-3, Co-	1.7×10^{0}	2.1×10^{-2}	30.4%	69.6%	0.95

Supplementary Table 17. Parameters for the fits of HDX-MS exchange kinetics curves of PA-1/3 copolymers using the bi-exponential function: $1 - \phi_{[PA17D]} = A_1 \cdot e^{-k_{fast} \cdot t} + A_2 \cdot e^{-k_{slow} \cdot t}$, where $A_1 + A_2 = 1$.

References

1. Scholtz, J. M., Grimsley, G. R. & Pace, C. N. Solvent Denaturation of Proteins and Interpretations of the m Value. in *Methods in Enzymology* vol. 466 549–565 (Academic Press, 2009).
2. Hunter, C. A. & Anderson, H. L. What is Cooperativity? *Angewandte Chemie International Edition* **48**, 7488–7499 (2009).
3. Li, Y. *et al.* Molecular basis of cooperativity in pH-triggered supramolecular self-assembly. *Nat Commun* **7**, 13214 (2016).
4. Lou, X. *et al.* Dynamic diversity of synthetic supramolecular polymers in water as revealed by hydrogen/deuterium exchange. *Nat Commun* **8**, 15420 (2017).
5. Wolffs, M. *et al.* Macroscopic Origin of Circular Dichroism Effects by Alignment of Self-Assembled Fibers in Solution. *Angew. Chem. Int. Ed.* **46**, 8203–8205 (2007).
6. Circular Dichroism Tips & Tricks for Biological Samples. *JASCO Inc.* https://jascoinc.com/learning-center/best-practices/circular-dichroism-tips-tricks/.
7. Korevaar, P. A. *et al.* Pathway complexity in supramolecular polymerization. *Nature* **481**, 492–496 (2012).
8. Korevaar, P. A., Schaefer, C., de Greef, T. F. A. & Meijer, E. W. Controlling Chemical Self-Assembly by Solvent-Dependent Dynamics. *J. Am. Chem. Soc.* **134**, 13482–13491 (2012).
